# Kinetic, computational, and phylogenetic characterisation of PA2125 from *Pseudomonas aeruginosa* PAO1: a hydroxybenzaldehyde dehydrogenase of the newly defined ALDH33 family

**DOI:** 10.1042/BSR20260546

**Published:** 2026-09-22

**Authors:** Gustavo A. Valero-Pibaral, Alexey Llopiz, Carlos A. Rueda-Sanabria, Héctor Riveros-Rosas, Arline Fernández-Silva, Carlos Mújica-Jiménez, Rosario A. Muñoz-Clares

**Affiliations:** 1 Departamento de Bioquímica, Facultad de Química, Universidad Nacional Autónoma de México, Ciudad de México, 04510, México; 2 Departamento de Bioquímica, Facultad de Medicina, Universidad Nacional Autónoma de México, Ciudad de México, 04510, México

**Keywords:** aldehyde dehydrogenase, gentisaldehyde, molecular dynamics, phylogenetic analysis, Pseudomonas aeruginosa, substrate inhibition

## Abstract

PA2125 from *Pseudomonas aeruginosa* PAO1 is annotated as an aldehyde dehydrogenase (ALDH), but its substrate specificity, catalytic properties, and phylogenetic family assignment had not been established. Here, we cloned the *PA2125* gene, produced and purified the recombinant PA2125 protein, and combined extensive substrate screening and steady-state kinetic characterisation with molecular dockings, molecular dynamics simulations, and phylogenetic analysis. PA2125 preferred NAD^+^ over NADP^+^, and screening of 54 aldehydes revealed a narrow substrate range centred on substituted benzaldehydes, particularly hydroxybenzaldehydes. Gentisaldehyde (GSA; 2,5-dihydroxybenzaldehyde) was the preferred substrate, with a *K*_M_ of 5.3 μM, a *k*_cat_ of 192 s^−1^, and a remarkably high *k*_cat_/*K*_M_ (3.54 × 10^7^ M^−1^ s^−1^). 3-Hydroxybenzaldehyde and 3,5-dihydroxybenzaldehyde also showed low *K*_M_ values but approximately sevenfold lower catalytic efficiencies. Initial-velocity patterns supported an ordered sequential steady-state Bi Bi mechanism in which NAD^+^ binds first. Whereas GSA showed no substrate inhibition at concentrations up to 45 × *K*_M_, the other two hydroxybenzaldehydes showed pronounced total substrate inhibition—likely due to aldehyde binding at the nucleotide-binding site—at concentrations close to their *K*_M_. Docking identified plausible interactions involving unusual active-site residues, whereas molecular dynamics simulations showed that GSA more frequently populated and more readily accessed catalytically competent geometries. Phylogenetic analyses defined a distinct bacterial ALDH family, here designated ALDH33, comprising three subfamilies, with PA2125 belonging to ALDH33A. Together, these results identify GSA as the preferred *in vitro* substrate of PA2125, support its biochemical assignment as a gentisaldehyde dehydrogenase, and provide a foundation for investigating its physiological role in aromatic-aldehyde metabolism.

## Introduction

Aldehyde dehydrogenases (ALDHs) constitute a highly diverse enzyme superfamily found across the three domains of life that catalyses the NAD(P)^+^-dependent oxidation of aldehydes. Depending on the mechanism of deacylation of the enzyme-acyl intermediate, the products are carboxylic acids (hydrolytic ALDHs), acyl phosphates (phosphorylating ALDHs), or acyl-CoA thioesters (CoA-dependent ALDHs). ALDHs share a conserved catalytic mechanism comprising two main steps, acylation and deacylation. In the acylation step, the strictly conserved catalytic cysteine residue acts as a nucleophile, attacking the aldehyde carbonyl carbon to form a thiohemiacetal intermediate; subsequent hydride transfer to NAD(P)^+^ generates NAD(P)H and a covalent thioacyl-enzyme intermediate. In hydrolytic ALDHs, the subsequent deacylation step involves a conserved glutamate residue that acts as a general base to activate a water molecule, which hydrolyses the thioacyl-enzyme intermediate to release the carboxylic acid product and regenerate the free enzyme [1,2]. A conserved asparagine residue forms part of the oxyanion hole, stabilising the negative charge that develops on the carbonyl oxygen in the tetrahedral intermediate formed during nucleophilic attack [2]. These three residues—the catalytic cysteine, the glutamate general base, and the asparagine—are conserved in hydrolytic ALDHs and are central to their catalytic mechanism.

ALDHs participate in biosynthetic and catabolic pathways and contribute to detoxification of endogenous and exogenous aldehydes. Despite their broad distribution and functional diversity, many bacterial ALDHs remain biochemically uncharacterised, and their physiological roles are frequently difficult to infer from sequence information alone because substrate specificity can be highly divergent even among close homologues. This problem is particularly relevant in metabolically versatile bacteria such as *Pseudomonas aeruginosa*, an environmental bacterium and opportunistic human pathogen of major clinical relevance owing to its intrinsic resistance to many antimicrobial agents and its capacity to cause severe nosocomial infections [3]. *P. aeruginosa* is notable for its metabolic versatility [4] and its capacity to degrade a wide range of xenobiotics, including aromatic compounds [5,6].

*P. aeruginosa strain* PAO1 encodes 23 ALDH enzymes [7]. Among them, PA2125 is annotated as an ALDH, but its substrate specificity, catalytic properties, and phylogenetic family assignment had not been experimentally established. To our knowledge, no close homologue of PA2125 has been biochemically characterised using purified recombinant protein. The ALDH enzyme from *Burkholderia* sp. strain BC1, encoded by *nolF*, which shares 69% identity to PA2125, was proposed to act as a gentisaldehyde dehydrogenase based on activity detected in crude extracts of cells grown on 2-hydroxy-1-naphthoic acid [8]. Similarly, the ALDH encoded by *psfA* in *Pseudomonas putida*, which shares 53% identity to PA2125, has been proposed to function as a furfural dehydrogenase because *psfA* expression was transcriptionally induced by furfuryl alcohol, furfural, or furoic acid [9]. These findings suggest that PA2125-like enzymes may participate in the oxidation of aromatic aldehydes, which may be intermediates in an unknown degradation pathway of environmental aromatic compounds of natural or anthropogenic origin. However, direct biochemical evidence and a mechanistic rationale for the substrate preference of this enzyme group are lacking.

Here, we report the first biochemical characterisation of PA2125 using the purified recombinant enzyme, complemented by molecular docking and molecular dynamics simulations, as well as comparative sequence and phylogenetic analyses. We cloned the *PA2125* gene, expressed the PA2125 protein in *Escherichia coli*, and performed a comprehensive kinetic characterisation to identify its preferred substrates *in vitro.* We also explored the molecular features contributing to aldehyde specificity using structural modelling, molecular docking, and molecular dynamics simulations and examined the evolutionary relationships of PA2125 within the ALDH superfamily through comparative sequence and phylogenetic analyses. These studies led us to propose a new ALDH family, which we designate ALDH33, and to characterise the conservation of active-site residues among its members. Our results establish that PA2125 is a hydroxybenzaldehyde dehydrogenase with particularly high catalytic efficiency toward gentisaldehyde, thereby providing a foundation for future studies aimed at defining its physiological role.

## Results and discussion

### Cloning and expression of the *PA2125* gene, and purification and molecular mass analysis of recombinant PA2125

The *PA2125* gene (NCBI Gene ID: 878590) was cloned from the *P. aeruginosa* PAO1 genome as described in the ‘Materials and methods’. The nucleotide sequence of the cloned gene was identical to the corresponding PAO1 reference sequence in the NCBI database. The open reading frame encodes a 482-residue polypeptide with a predicted molecular mass of 51.4 kDa for the untagged monomer (52.2 kDa including the N-terminal His tag calculated from its amino acid sequence using ExPASy ProtParam [10]). Sodium dodecyl sulfate–polyacrylamide gel electrophoresis (SDS–PAGE) analysis of the purified recombinant protein showed a single band at ∼54 kDa (Supplementary Figure S1A), consistent with the expected size of the His-tagged monomer. When analysed by size-exclusion chromatography, PA2125 eluted as a single symmetric peak (Supplementary Figure S1B) with an apparent molecular mass (∼171 kDa) intermediate between those expected for a dimer and a tetramer and closer to a trimeric value. However, a trimeric assembly is not a structurally plausible oligomeric state for canonical ALDHs, whose quaternary organisation is based on subunit interfaces that generate dimers and, in many cases, tetramers formed as dimers of dimers. Therefore, the SEC result is more reasonably interpreted as reflecting the anomalous hydrodynamic behaviour of a tetrameric assembly than as evidence for a trimer. This interpretation is supported by the close correspondence between the elution volume of PA2125 and those of other tetrameric *P. aeruginosa* PAO1 ALDHs characterised under identical conditions [11,12]. In addition, native PAGE (data not shown) showed a single predominant migrating species, with no resolved second band suggestive of coexisting stable dimeric and tetrameric populations. Because SEC and native PAGE do not provide an unambiguous determination of molar mass, approaches such as SEC-MALS or SAXS would be required to establish the oligomeric state definitively.

### Cofactor specificity analysis identifies NAD^+^ as the preferred cofactor of PA2125

**Figure 1:**
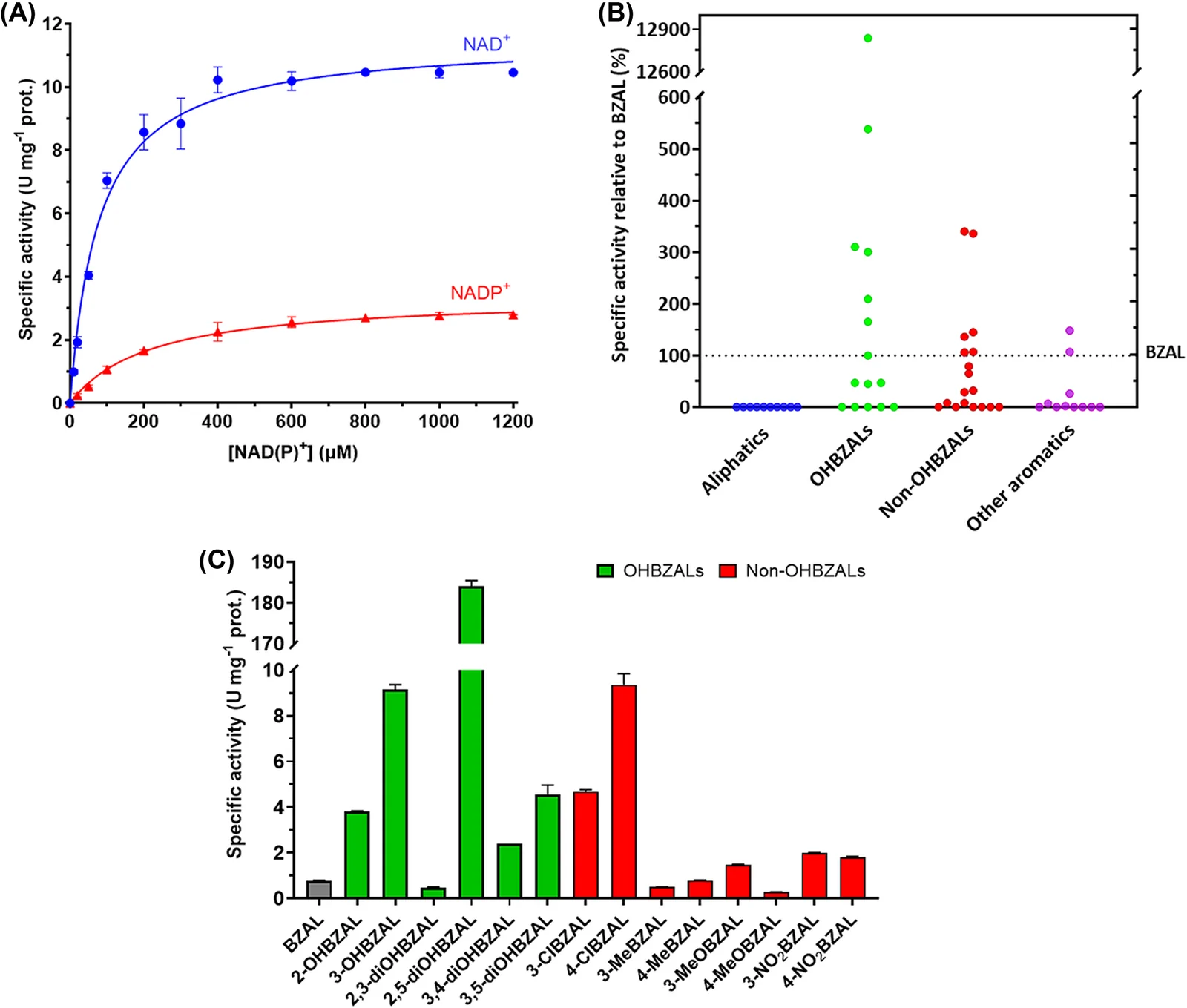
Cofactor and aldehyde substrate preference of PA2125 (**A**) Saturation curves with NAD^+^ and NADP^+^ at 10 mM BZAL. Data are means ± SD of three independent experiments, and lines are fits to the Michaelis–Menten equation. (**B**) Specific activities with the aldehydes tested, expressed relative to the activity with BZAL (100%; dotted line). Assays were performed with 0.6 mM NAD^+^ and 1 mM aldehyde, except for OHBZALs, which were assayed at 0.25 mM. Each data point represents a single substrate. The y-axis is broken to accommodate the much higher activity observed with GSA. (**C**) Specific activities with selected OHBZALs and non-OHBZAL derivatives at 0.1 mM aldehyde and otherwise under the conditions described for panel B. Data are means ± SD of at least two independent experiments; the y-axis is broken as indicated.

Initial velocity data obtained at pH 8.0, fixed 10 mM benzaldehyde—which is the reference substrate used in our studies—and nucleotide concentration varied from 0 to 1.2 mM indicated that NAD^+^ is a better substrate than NADP^+^ (Figure 1A). The estimated apparent kinetic parameters for NAD^+^ were *V*_max_ = 11.5 ± 0.2 U mg prot.^−1^, *k*_cat_ = 10.0 ± 0.2 s^−1^, *K*_M_ = 78.2 ± 6.5 μM, and *k*_cat_/*K*_M_ = (1.28 ± 0.09) × 10^5^ M^−1^ s^−1^. For NADP^+^ the estimated apparent kinetic parameters were *V*_max_ = 3.39 ± 0.09 U mg prot.^−1^, *k*_cat_ = 2.95 ± 0.08 s^−1^, *K*_M_ = 215 ± 19 μM, and *k*_cat_/*K*_M_ = (1.37 ± 0.09) × 10^4^ M^−1^ s^−1^. Because these parameters were determined using BZAL, a comparatively poor substrate for PA2125 as shown below, they should be regarded as apparent values specific to this experimental context, valid for comparing NAD^+^ and NADP^+^ with each other under identical conditions, but not directly comparable to the true *K*_A_ and *k*_cat_/*K*_A_ values reported later for the preferred substrate, GSA. The ten-times higher *k*_cat_/*K*_M_^NAD+^ value than *k*_cat_/*K*_M_^NADP+^ suggests that NAD^+^ is probably the cofactor used *in vivo* by PA2125. Although the intracellular NAD^+^ and NADP^+^ concentrations have not, to our knowledge, been reported for *P. aeruginosa*, the general observation that free NADP^+^ pools are typically considerably smaller than NAD^+^ pools would reinforce the kinetic preference for NAD^+^. Nevertheless, the measurable activity with NADP^+^ does not allow us to exclude a broader cofactor specificity and a physiological role for NADP^+^.

### Aldehyde specificity analysis identifies hydroxybenzaldehydes as the preferred substrates

Sequence comparison of PA2125 with related hydrolytic ALDHs revealed unusual substitutions in the aldehyde-binding site, including replacement of residues that are typically aromatic by polar or charged residues, as well as the presence of an aromatic residue at a position that is usually non-aromatic. In PA2125, the aldehyde-binding region contains unusual substitutions at four positions that are generally conserved among ALDHs. Ser150, Arg157, and Glu449 occupy positions that are frequently aromatic in other ALDHs (equivalent to Phe170, Trp177, and Phe465 in human ALDH2 numbering, respectively), and are instead polar or charged in PA2125; in contrast, Phe281 occupies a position that is typically non-aromatic (equivalent to Cys301 in ALDH2), but is aromatic in PA2125. These features suggested that PA2125 might exhibit a distinct aldehyde specificity. To define its substrate preferences, we carried out a comprehensive analysis of PA2125 substrate specificity by screening a broad set of aldehydes (54 in total) representing distinct chemical classes: aliphatic aldehydes, benzaldehyde derivatives, and other aromatic or heteroaromatic aldehydes. In the present study, benzaldehyde (BZAL) was chosen as the reference compound for comparing PA2125 activity toward the aldehydes tested. Because when tested under our assay conditions several BZAL derivatives, particularly hydroxyBZALs (OHBZALs) and some hydroxybenzoates, exhibited important absorbance at 340 nm (Supplementary Figure S2), their contributions to the measured absorbance were taken into account. This absorbance mainly arises from partial deprotonation at pH 8.0 and the resulting substituent-dependent changes in their electronic delocalisation [13]. Therefore, the molar absorptivity of these compounds was determined experimentally under our assay conditions, and the results are shown in Supplementary Table S1. These values were used to calculate the corrected molar absorptivity at 340 nm (ε_340_,_corr_) used to determine PA2125 activity with these aldehydes, as described in the ‘Materials and methods’ section.

The results of the aldehyde screening (Figure 1B and Supplementary Table S2) showed that PA2125 displayed negligible activity toward the aliphatic aldehydes tested, indicating that it is not a broad-spectrum aldehyde dehydrogenase. The poor activity observed with these aldehydes, despite the intrinsically greater reactivity of their carbonyl groups, indicates that PA2125 activity does not depend on carbonyl electrophilicity alone, but also on how the aldehyde binds and is positioned within the active site, affecting steps beyond the initial nucleophilic attack by the catalytic cysteine (Cys282). BZAL and its derivatives were clearly preferred, indicating that PA2125 recognises aromatic aldehydes much more efficiently than aliphatic ones. However, the very low activities observed with phenylacetaldehyde and 3-phenylpropanal show that the presence of an aromatic ring is not sufficient for efficient catalysis. Instead, PA2125 favours BZAL-type substrates in which the aldehyde group is directly attached to the aromatic ring. This requirement may reflect the limited rotational freedom of the aldehyde group in BZAL derivatives, resulting from its conjugation with the benzene π system, which favours a relatively fixed orientation of the carbonyl group with respect to the aromatic ring. Non-BZAL aromatic and heteroaromatic aldehydes were generally poor substrates. It is of note that furfural showed negligible activity (2% relative to BZAL), and the related 5-hydroxymethylfurfural, although retaining comparatively higher activity (26%), is still a poor substrate, arguing against PA2125 functioning as a furfural dehydrogenase, different from the *P. putida* PsfA enzyme.

Among the BZAL derivatives, the best PA2125 substrates were found among the hydroxylated benzaldehydes (OHBZALs), several of which greatly exceeded the activity of any non-hydroxylated derivative (non-OHBZALs). This preference for hydroxylation is not absolute, however, as some OHBZALs (2,3,4-triOHBZAL and 2,4,6-triOHBZAL) showed negligible activity, comparable to the poorest non-OHBZALs. 2,5-diOHBZAL (gentisaldehyde, GSA) stands out as an exceptionally good substrate, followed by 3-OHBZAL, 3,5-diOHBZAL, and 2,4,5-triOHBZAL. Because hydroxyl substituents donate electron density to the aromatic ring, this feature is unlikely to result from a simple increase in the intrinsic electrophilicity of the aldehyde carbonyl. Instead, it more likely points to favourable binding interactions, such as hydrogen bonding and/or improved positioning of the aromatic ring within the active site. The analysis of the non-OHBZALs showed that these compounds were, overall, poorer substrates than the OHBZALs, although several were still substantially better substrates than the aliphatic aldehydes and BZAL itself. In this group, chloro- and nitroBZALs gave relatively high activities. For these substrates, electron-withdrawing substituents may contribute positively by increasing the electrophilicity of the aldehyde carbonyl, provided that steric and positional constraints are satisfied.

Among aromatic aldehydes, including heteroaromatic, PA2125 activity was strongly influenced by the identity and position of ring substituents, with a general preference for 3- and 4-substituted compounds over the corresponding 2-substituted analogues. This preference suggests that substitution adjacent to the aldehyde group is strongly unfavourable for productive catalysis, likely because of steric interference and/or suboptimal orientation of the substrate within the active site. However, the effect of substitution at position 2 was not uniformly unfavourable. GSA was by far the best substrate tested; 2,4,5-triOHBZAL had a *k*_cat_/*K*_M_ about 60-fold higher than BZAL, and 2-OHBZAL (salicylaldehyde) was a much better substrate than other ortho-substituted BZALs. This indicates that PA2125 does not simply disfavour ortho substitution; rather, substrate recognition depends on the chemical nature of the substituent at position 2 and on the overall substitution pattern of the aromatic ring. In this context, the GSA and 2,3,5-triOHBZAL behaviour suggests that the shared 2,5-dihydroxy substitution pattern is particularly well accommodated in the active site. The ortho-hydroxyl group may contribute by preorganising the aldehyde through intramolecular hydrogen bonding, whereas the additional hydroxyl group at position 5 may provide favourable interactions that compensate for, or even reverse, the unfavourable effects observed with other 2-substituted derivatives. Similarly, the relatively high activity of salicylaldehyde may reflect analogous intramolecular hydrogen bonding between the ortho-hydroxy group and the aldehyde oxygen, favouring a preorganised conformation, partially offsetting the steric penalty associated with substitution adjacent to the aldehyde group and facilitating productive positioning in the active site.

Although BZAL derivatives bearing substituents at position 3 or 4 were generally better substrates than their corresponding 2-derivatives, 4-OHBZAL was a clear exception to this trend since it was a very poor PA2125 substrate. To determine whether this low activity reflected poor binding or, instead, catalytically non-productive binding, we examined the effect of 4-OHBZAL on the oxidation of 2-OHBZAL. Initial rates were measured by following the decrease in absorbance at 400 nm, a wavelength at which NADH contributes negligibly, whereas 2-OHBZAL absorbs sufficiently to allow substrate consumption to be monitored directly. Increasing concentrations of 4-OHBZAL caused a clear decrease in the rate of 2-OHBZAL oxidation (Supplementary Figure S3A), but the experiment does not by itself define the inhibition mechanism or provide a *K*_i_ value. Docking simulations using the deprotonated 4-OHBZAL species, which predominates at pH 8.0 (C4-OH theoretical p*K*_a_ = 7.32), suggested its binding in the aldehyde-binding site but in a catalytically incompetent orientation apparently stabilised by interaction of the negatively charged hydroxyl group with the Arg157 side-chain (Supplementary Figure S3B). Thus, the poor catalytic efficiency of PA2125 with 4-OHBZAL does not appear to result from lack of binding, but rather from non-productive binding within the active site.

Because the initial screening identified several BZAL derivatives that were oxidised more efficiently than unsubstituted BZAL and given that substrate inhibition is frequently observed in hydrolytic ALDHs [14], we further compared this subset at a lower aldehyde concentration (0.1 mM). We assume this concentration to be closer to the low levels expected for reactive aldehydes under steady-state intracellular conditions, as these compounds are typically converted rapidly to their more stable carboxylic acid products. The results (Figure 1C) support the conclusion that PA2125 preferentially oxidises OHBZALs.

### Saturation kinetics of PA2125 with selected benzaldehyde derivatives

**Figure 2:**
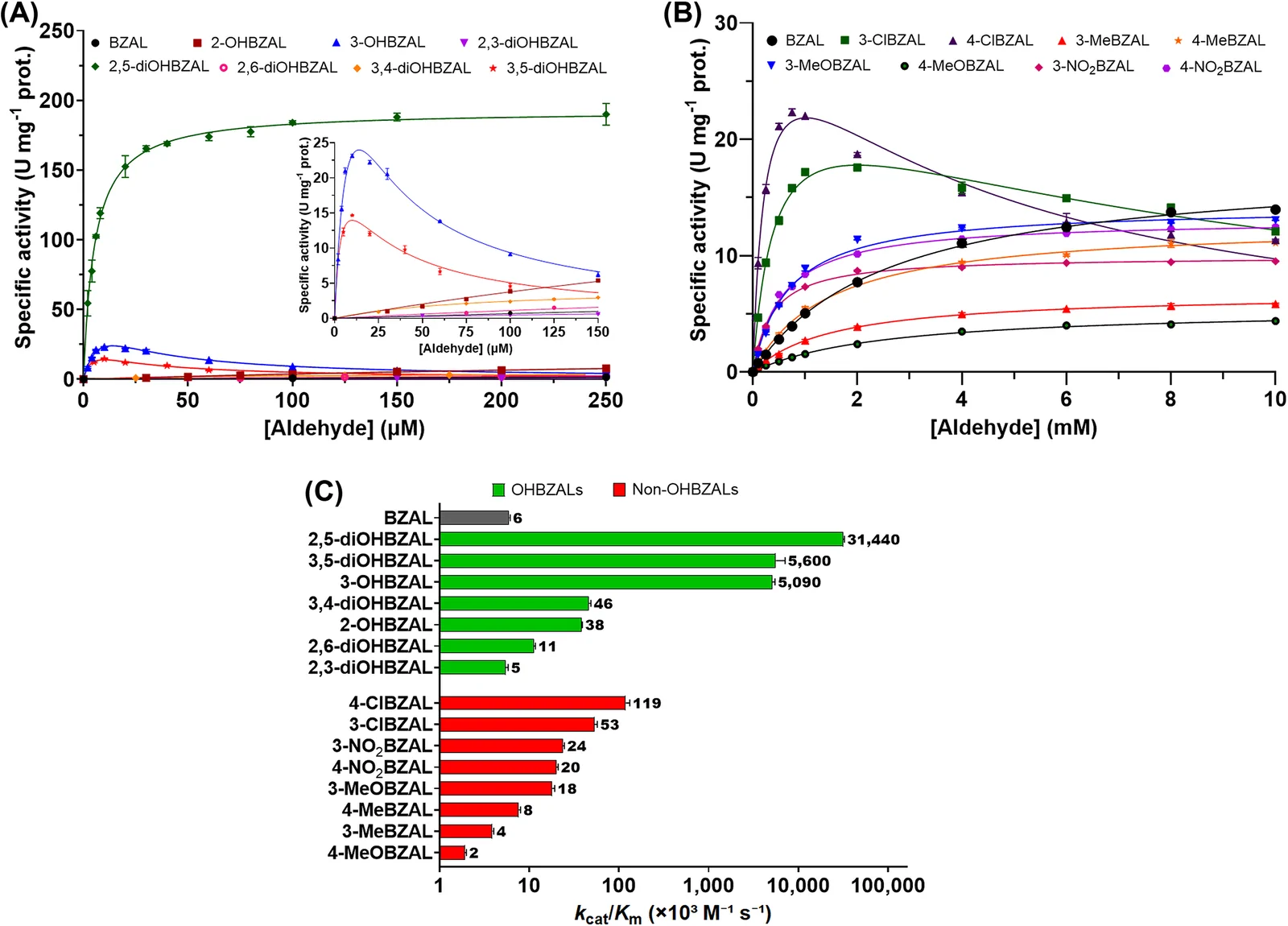
Saturation kinetics of PA2125 with BZAL derivatives Saturation curves with OHBZALs (**A**) and non-OHBZALs (**B**). The inset in panel (A) shows an expanded view of the low-activity substrates, excluding GSA. Data are means ± SD from assays performed with at least two independent enzyme preparations. Enzyme assays were carried out at 30°C in 100 mM phosphate buffer, pH 8.0, containing 0.6 mM NAD^+^ and variable concentrations of the indicated aldehydes. Initial-velocity values were determined from the linear increase in absorbance at 340 nm. Where necessary, rates were calculated using corrected apparent molar absorptivity values that account for the absorbance of the aldehyde and/or reaction product, as described in the ‘Materials and methods’ section and listed in Supplementary Table S1. Lines represent the best individual nonlinear fits to the Michaelis–Menten or substrate-inhibition equations (eqns 2 and 3), as appropriate. R^2^ values for each fit are given in Table 1. (**C**) Comparison of the corresponding apparent *k*_cat_/*K*_M_ values. Full compound names and abbreviations are given in Supplementary Table S2.

To obtain a complete kinetic description of PA2125 substrate specificity, saturation kinetics experiments were performed with BZAL and selected BZAL derivatives. The results confirmed that OHBZALs are the best *in vitro* PA2125 substrates (Figure 2A and Table 1). The best one was GSA, which exhibits very high apparent *k*_cat_ (168 ± 1 s^−1^)—estimated per active site—and very low apparent *K*_M_ (5.3 ± 0.2 μM) values. To the best of our knowledge, the PA2125 apparent *k*_cat_/*K*_M_ (3.54 × 10^7^ M^−1^ s^−1^) value with this aldehyde is among the highest reported for a hydrolytic ALDH, exceeding by about fourfold the highest value reported for a bacterial aromatic aldehyde dehydrogenase, LigV (vanillin dehydrogenase from *Sphingobium* sp. SYK-6; *k*_cat_/*K*_M_ = 8.8 × 10^6^ M^−1^ s^−1^) [15], and lying within less than an order of magnitude of the diffusion-controlled limit for enzyme catalysis (10^8^–10^9^ M^−1^ s^−1^). In addition, GSA does not inhibit PA2125, while the other two PA2125 second-best substrates—3-OHBZAL and 3,5-diOHBZAL, which have a very low *K*_M,app_, similar to that of GSA—exhibit very strong substrate inhibition. This pattern is unusual in two respects. First, substrate inhibition, which appears to be a property of many, if not all, the hydrolytic ALDHs [14], is generally observed only at substrate concentrations well above their *K*_M_ values, whereas inhibition by 3-OHBZAL and 3,5-diOHBZAL occurred near *K*_M,app_. Second, despite the broad concentration range examined, GSA did not inhibit PA2125 even at concentrations greatly exceeding its *K*_M,app_. Thus, GSA is distinguished not only by its high catalytic efficiency but also by the absence of the substrate inhibition displayed by 3-OHBZAL and 3,5-diOHBZAL, the two other hydroxybenzaldehydes oxidised with the highest catalytic efficiencies, which exhibited *K*_M_ values comparable to that of GSA.

**Table 1:** Apparent kinetic parameters of PA2125 with BZAL and its hydroxylated and non-hydroxylated derivatives

Substrate	*k*_cat_ (s^−1^)	*K*_M_ (μM)	*k*_cat_/*K*_M_ (×10^3^ M^−1^ s^−1^)	*K*_IS_ (μM)	R^2^
BZAL	15.56 ± 0.20	2,593 ± 90	6.00 ± 0.14	NA	0.9993
**Hydroxylated BZAL derivatives**					
2-OHBZAL	22.10 ± 0.27	576 ± 16	38.39 ± 0.67	NA	0.9991
3-OHBZAL	50.6 ± 6.1	9.9 ± 1.9	5.090 ± 380	19.5 ± 3.4	0.9933
2,3-diOHBZAL	1.52 ± 0.05	278 ± 26	5.45 ± 0.37	NA	0.9912
2,5-diOHBZAL (GSA)	167.6 ± 1.1	5.33 ± 0.18	31,440 ± 940	NA	0.9983
2,6-diOHBZAL	6.84 ± 0.28	607 ± 49	11.26 ± 0.47	NA	0.9961
3,4-diOHBZAL	3.91 ± 0.12	84.2 ± 6.8	46.4 ± 2.4	NA	0.9962
3,5-diOHBZAL	21.4 ± 3.6	3.8 ± 1.7	5,600 ± 1,500	26.2 ± 7.0	0.9877
**Nonhydroxylated BZAL derivatives**					
3-ClBZAL	21.7 ± 1.0	406 ± 46	53.5 ± 3.9	9,900 ± 1,200	0.9940
4-ClBZAL	27.7 ± 1.8	233 ± 39	119 ± 13	4,430 ± 650	0.9835
3-MeBZAL	5.89 ± 0.07	1,511 ± 61	3.90 ± 0.12	NA	0.9991
4-MeBZAL	11.17 ± 0.21	1,460 ± 91	7.65 ± 0.36	NA	0.9969
3-MeOBZAL	12.36 ± 0.25	684 ± 55	18.1 ± 1.2	NA	0.9936
4-MeOBZAL	4.77 ± 0.09	2,480 ± 130	1.925 ± 0.069	NA	0.9984
3-NO_2_BZAL	8.60 ± 0.08	364 ± 16	23.73 ± 0.92	NA	0.9974
4-NO_2_BZAL	11.39 ± 0.12	563 ± 25	20.22 ± 0.76	NA	0.9971

Among the triOHBZALs tested, only 2,4,5-triOHBZAL was efficiently oxidised by PA2125, with an apparent *k*_cat_/*K*_M_ value approximately one order of magnitude lower than those of 3-OHBZAL and 3,5-diOHBZAL and nearly two orders of magnitude lower than that of GSA. The difference relative to 3-OHBZAL and 3,5-diOHBZAL arose mainly from the higher *K*_M,app_ of 2,4,5-triOHBZAL, whereas its lower efficiency relative to GSA reflected both an approximately 12-fold higher *K*_M,app_ and an approximately eight-fold lower *k*_cat,app_. The substantial catalytic efficiency of 2,4,5-triOHBZAL is noteworthy because this compound retains the 2,5-dihydroxy substitution pattern present in GSA. As discussed above, the hydroxyl groups at C2 and C5 may favour productive substrate accommodation and positioning of the aldehyde group, whereas the additional hydroxyl group at C4 markedly decreases catalytic efficiency, probably for reasons similar to those of 4-OHBZAL, given that at the assay pH, 8.0, the hydroxyl group at position 4 is predominantly deprotonated (C4-OH theoretical p*K*_a_ = 7.53). The resulting negative charge may impair productive binding through unfavourable interactions or suboptimal accommodation within the active site, potentially contributing to the higher *K*_M_ and the lower *k*_cat_/*K*_M_ of 2,4,5-triOHBZAL relative to GSA. No substrate inhibition by 2,4,5-triOHBZAL was detected within the experimentally accessible concentration range. However, because its high absorbance limited the highest concentration assayed to only 2.25 times *K*_M_, inhibition at higher concentrations cannot be excluded. Nevertheless, this behaviour contrasts with that of 3-OHBZAL and 3,5-diOHBZAL, which showed pronounced substrate inhibition at concentrations close to their respective *K*_M_ values despite their higher catalytic efficiencies.

Non-OHBZALs exhibited higher apparent Michaelis–Menten constant and lower maximal activities than those of the best OHBZAL substrates (Figure 2B and Table 1). The chlorinated derivatives were the most active within this group, particularly 4-ClBZAL, although both chloro-substituted aldehydes required millimolar concentrations to approach saturation and showed substrate inhibition. BZAL, 3-MeOBZAL, 4-MeBZAL, and 4-NO_2_BZAL behaved as moderate substrates, whereas 4-MeOBZAL and 3-MeBZAL showed low activity. Thus, although PA2125 can oxidize several non-OHBZALs, their kinetic behaviour reinforces the marked preference of the enzyme for OHBZALs, especially GSA.

Comparison of the apparent specificity constants further emphasised the marked substrate preference of PA2125 for a restricted subset of OHBZALs (Figure 2C and Table 1). The highest apparent *k*_cat_/*K*_M_ value was obtained with 2,5-diOHBZAL, followed by 3,5-diOHBZAL and 3-OHBZAL. The catalytic efficiency of these three substrates clearly separated them from the remaining hydroxy and non-hydroxy BZAL derivatives.

### Steady-state kinetic and product inhibition analyses of GSA, 3-OHBZAL and 3,5-diOHBZAL shed light on the kinetic mechanism of PA2125 and its substrate inhibition

**Figure 3:**
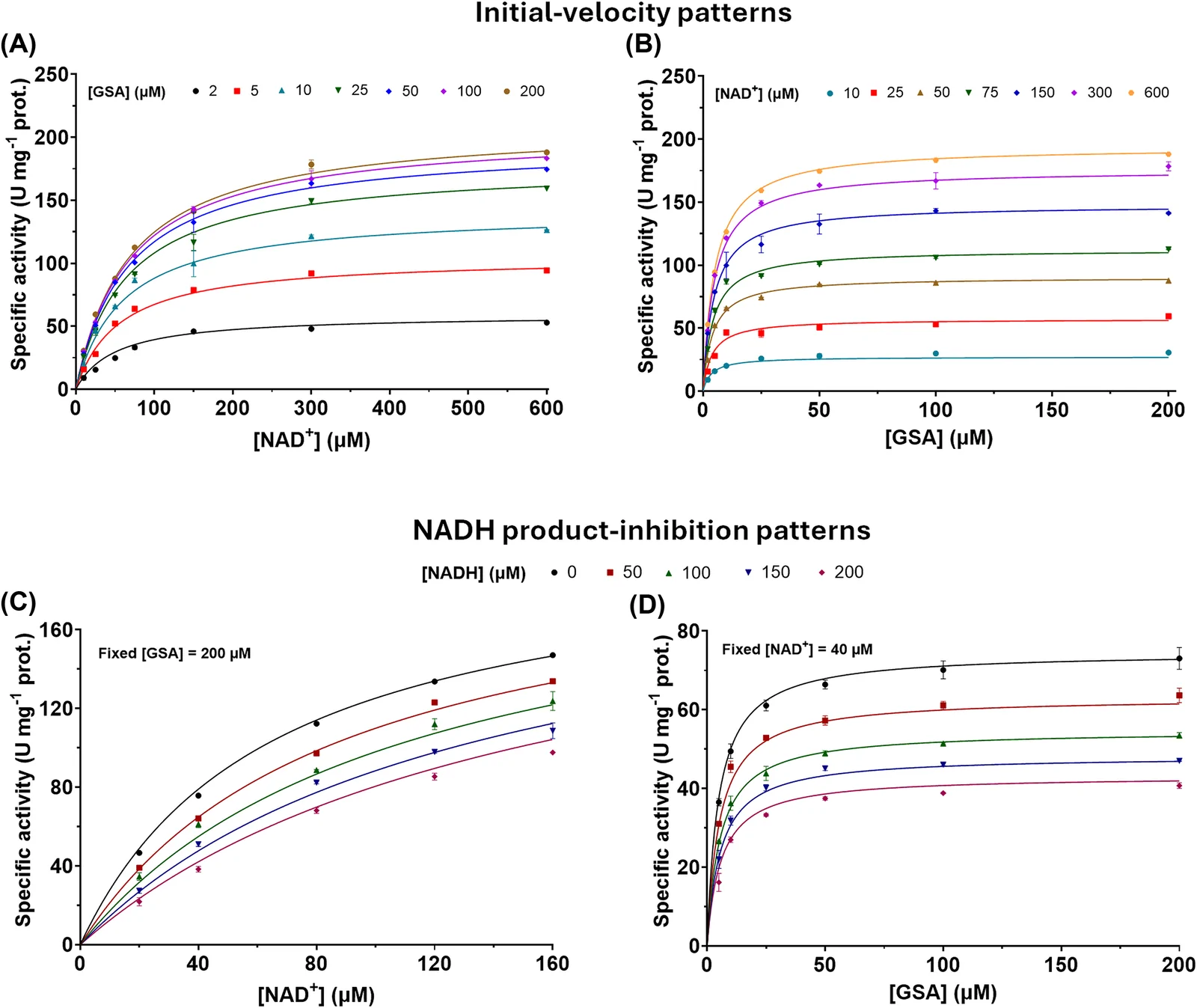
Initial-velocity and NADH product-inhibition patterns of PA2125 with GSA and NAD^+^ Initial velocities were measured by varying NAD^+^ at several fixed GSA concentrations (**A**) or GSA at several fixed NAD^+^ concentrations (**B**). NADH product inhibition was analysed by varying NAD^+^ at fixed saturating GSA (**C**) or GSA at fixed nonsaturating NAD^+^ (**D**) at the indicated NADH concentrations. Lines in panels (A) and (B) are global nonlinear fits to an ordered sequential steady-state Bi Bi mechanism (eqn 5). Lines in panels (C) and (D) are fits to competitive or mixed-inhibition equations (eqns 7 and 8, respectively). R^2^ values for the global fit to (eqn 5) are 0.9959, and for fits to (eqns 7 and 8) are 0.9924 and 0.9895, respectively. Data are means ± SD of two to three independent experiments. Other assay conditions and data treatment were as described in the legend to Figure 2.

To investigate the kinetic mechanism of PA2125 with GSA, initial-velocity patterns were obtained by varying either NAD^+^ or GSA at several fixed concentrations of the cosubstrate. The initial-velocity data were consistent with a sequential Bi Bi mechanism, either ordered or rapid-equilibrium random (Figure 3A,B), which was further distinguished by product-inhibition studies. NADH was competitive with respect to NAD^+^ at a fixed saturating GSA concentration and mixed with respect to GSA at a fixed subsaturating NAD^+^ concentration (Figure 3C,D). Together, these patterns support an ordered sequential Bi Bi mechanism in which NAD^+^ binds first and NADH is the last product released. The estimated kinetic parameters are given in Table 2, and the corresponding Lineweaver-Burk plots are shown in Supplementary Figure S4.

**Table 2:** Steady-state kinetic parameters of PA2125 for NAD^+^ and GSA, and constants for inhibition by the product NADH

Kinetic parameter	Estimated value	
**Initial-velocity analysis** ^a^		
*V*_max_ (U mg^−1^ prot.)	216.3 ± 2.6	
*K*_iA_ (μM)	43.0 ± 8.0	
*K*_A_ (μM)	69.6 ± 2.5	
*K*_B_ (μM)	5.31 ± 0.28	
*k*_cat_ (s^−1^)	188.1 ± 2.3	
*k*_cat_/*K*_A_ (×10^6^ M^−1^ s^−1^)	2.70 ± 0.08	
*k*_cat_/*K*_B_ (×10^7^ M^−1^ s^−1^)	3.54 ± 0.16	
**NADH product-inhibition analysis** ^b^		
Varied substrate	NAD^+^	GSA
Fixed substrate	GSA^c^	NAD^+^
*V*_max_ (U mg^−1^ prot.)	205.3 ± 6.2	74.59 ± 0.96
*K*_M_ (μM)	64.0 ± 4.9	4.91 ± 0.35
*K*_ic_ (μM)	140 ± 11	186 ± 38
*K*_ii_ (μM)	NA	275 ± 15
Type of inhibition	Competitive	Mixed

a Estimated parameters ± standard errors were obtained by global nonlinear fitting of the initial velocity data shown in Figure 3A,B to the equation for an ordered sequential steady-state Bi Bi mechanism (eqn 5).

b Estimated parameters ± standard errors were obtained by individual nonlinear fitting of the NADH product-inhibition data shown in Figure 3C,D to the equations for competitive or mixed inhibition (eqns 7 and 8, respectively).

c GSA was fixed at 250 μM, a saturating concentration; therefore, the estimated kinetic parameters could be considered true parameters.

The *K*_iA_ value of 43 μM indicates appreciable affinity of PA2125 for NAD^+^, and the similar *K*_A_ value suggests that when GSA is saturating, PA2125 reaches kinetic saturation with NAD^+^ at concentrations close to those required for NAD^+^ binding to the free enzyme. Thus, the aldehyde-dependent steps do not appear to impose an additional kinetic requirement for NAD^+^ under these conditions. This, together with *K*_B_ value in the low micromolar range, *k*_cat_ close to 200 s^−1^, *k*_cat_/*K*_B_ in the 10^7^ M^−1^ s^−1^ range, and the absence of substrate inhibition, indicates that GSA is efficiently oxidised once the enzyme NAD^+^ complex has formed. The catalytic efficiency with respect to NAD^+^ was approximately one order of magnitude lower than that observed with GSA, mainly reflecting the much lower *K*_B_ value of GSA. As *K*_ic_ can be interpreted as the dissociation constant of NADH from the E⋅NADH complex, its value (140 μM), approximately three-fold higher than the *K*_iA_ for NAD^+^ (43 μM), indicates preferential binding of the oxidised cofactor over the reduced one, while still allowing product inhibition at sufficiently high NADH concentrations. Thus, as NAD^+^ binding is the first step in the ordered mechanism, changes in the cellular NAD^+^/NADH ratio could modulate PA2125 activity by affecting formation of the enzyme-NAD^+^ complex.

**Figure 4:**
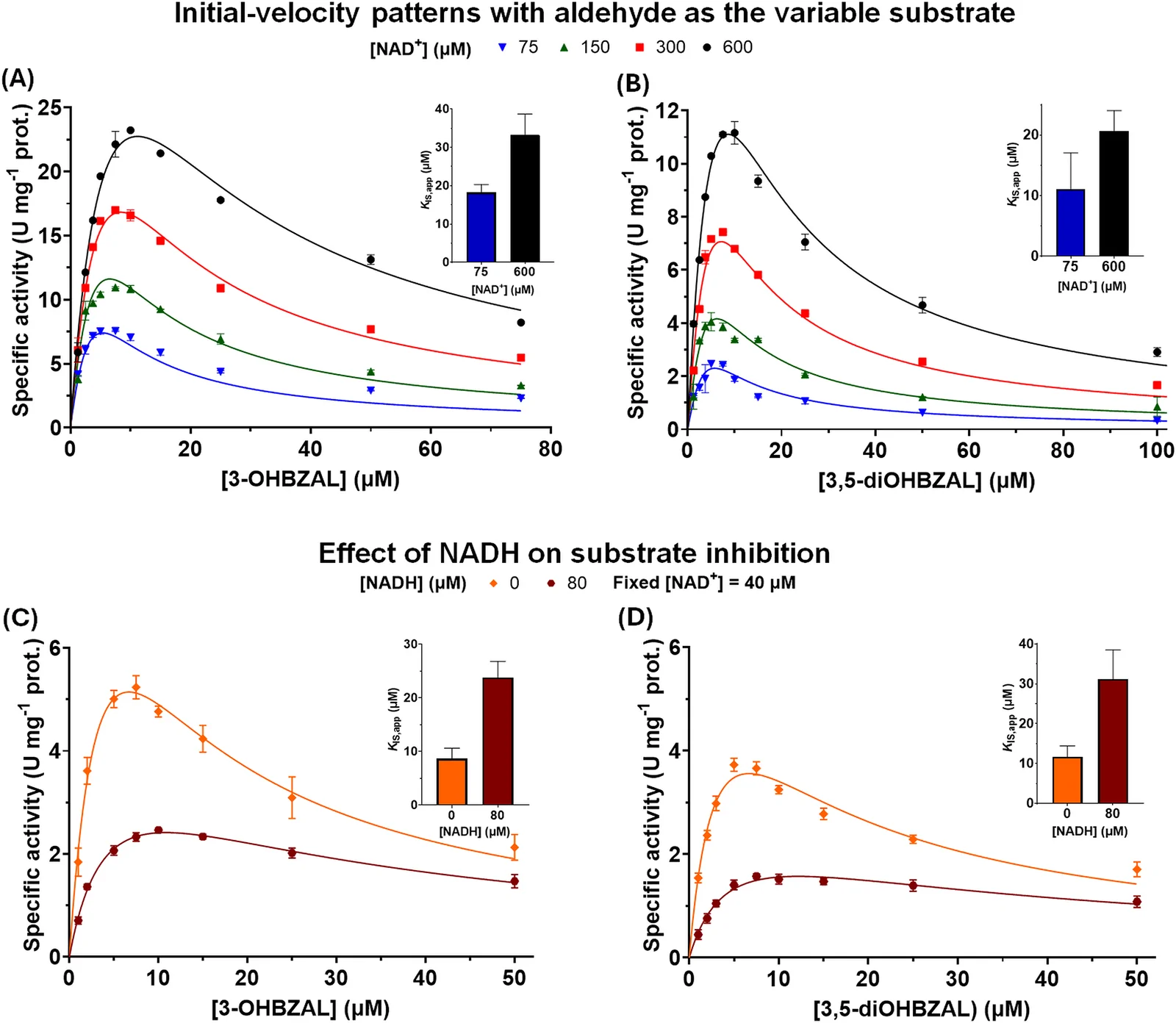
Initial-velocity patterns of PA2125 with 3-OHBZAL and 3,5-diOHBZAL and effect of NADH on substrate inhibition by these aldehydes Initial velocities were measured by varying 3-OHBZAL (**A**) or 3,5-diOHBZAL (**B**) at the indicated fixed NAD^+^ concentrations. Lines are global nonlinear fits to an ordered sequential Bi Bi equation incorporating total substrate inhibition (eqn 6). R^2^ values for the global fit to (eqn 6) are 0.9844 for 3-OHBZAL and 0.9859 for 3,5-diOHBZAL. Insets show the estimated *K*_IS,app_ values for the lowest and highest NAD^+^ concentrations tested. The effects of 80 μM NADH on substrate inhibition by 3-OHBZAL (**C**) and 3,5-diOHBZAL (**D**) were examined at 40 μM NAD^+^. Lines are individual fits to the Michaelis–Menten substrate-inhibition equation (eqn 3). R^2^ values are 0.9629 and 0.9844 for 3-OHBZAL at 0 and 80 μM NADH, respectively, and 0.9397 and 0.9485 for 3,5-diOHBZAL at 0 and 80 μM NADH, respectively. Insets show the *K*_IS_,_app_ values estimated from each curve. In all panels, data are means ± SD of at least three independent experiments.

Because substrate inhibition can complicate the analysis of initial-velocity patterns and the estimation of steady-state kinetic parameters [14], we further examined PA2125 kinetics with its two next-best substrates, 3-OHBZAL and 3,5-diOHBZAL, both of which, unlike GSA, showed strong substrate inhibition. The results of this analysis are shown in Figure 4 and Table 3.

**Table 3:** Steady-state kinetic parameters for the reaction of PA2125 with 3-OHBZAL and 3,5-diOHBZAL and the effect of NADH on substrate inhibition by these aldehydes

Kinetic parameter	3-OHBZAL		3,5-diOHBZAL	
**Initial-velocity analysis** ^a^				
*V*_max_ (U mg prot.^-1^)	66.4 ± 4.5		65.2 ± 8.5	
*K*_A_ (μM)	83.4 ± 9.1		337 ± 36	
*K*_B_ (μM)	8.7 ± 1.0		11.9 ± 2.1	
*K*_IS_ (μM)	1.7 ± 0.3		2.3 ± 0.5	
**Substrate-inhibition analysis** ^b^				
[NADH] (μM)	0	80	0	80
*V*_max,app_ (U mg prot.^-1^)	13.1 ± 2.0	4.6 ± 0.3	7.6 ± 1.1	2.8 ± 0.3
*K*_B,app_ (μM)	5.2 ± 1.2	4.9 ± 0.6	3.8 ± 0.9	4.7 ± 0.9
*K*_IS,app_ (μM)	8.7 ± 2.0	23.8 ± 3.0	11.7 ± 2.7	31.2 ± 7.4

a Values are parameter estimates ± standard errors obtained by global nonlinear fitting of the initial-velocity data shown in Figure 4A,B to the equation for an ordered sequential steady-state Bi Bi mechanism with total substrate inhibition by the aldehyde (eqn 6) and *K*_iA_ constrained to equal *K*_A_.

b Apparent parameter estimates ± standard errors were obtained by individual nonlinear fits of the aldehyde-saturation data shown in Figure 4C,D to the equation of Michaelis–Menten modified to account for total substrate inhibition by the aldehyde (eqn 3).

When each aldehyde-saturation curve was fitted individually to the equation for total substrate inhibition (eqn 3; these fits are not shown), i.e. inhibition in which velocity approaches zero, rather than plateauing, at high substrate concentrations, apparent *K*_IS_ values were obtained at each fixed NAD^+^ concentration and found to increase with NAD^+^ concentration, suggesting that NAD^+^ antagonises inhibition by the aldehyde. We therefore interpret the substrate inhibition as arising from nonproductive binding of the aldehyde to, or near, the nicotinamide nucleotide-binding site, thereby interfering with formation of the catalytically competent enzyme·NAD^+^ complex. Consistent with this interpretation, the data were globally fitted to the equation for an ordered sequential steady-state Bi Bi mechanism incorporating total substrate inhibition by the aldehyde (eqn 6). To obtain a good fit, *K*_iA_ was constrained to equal *K*_A_, consistent with the behaviour observed with GSA; an extra sum-of-squares F-test for nested models confirmed that the additional free parameter did not significantly improve the fit for either aldehyde (*P* > 0.19 with both aldehydes). Under this constraint, the data were reasonably described by the same ordered sequential kinetic mechanism deduced for GSA (Figure 4A,B and Table 3), with the main difference being the additional substrate-inhibition term required for 3-OHBZAL and 3,5-diOHBZAL.

**Figure 5:**
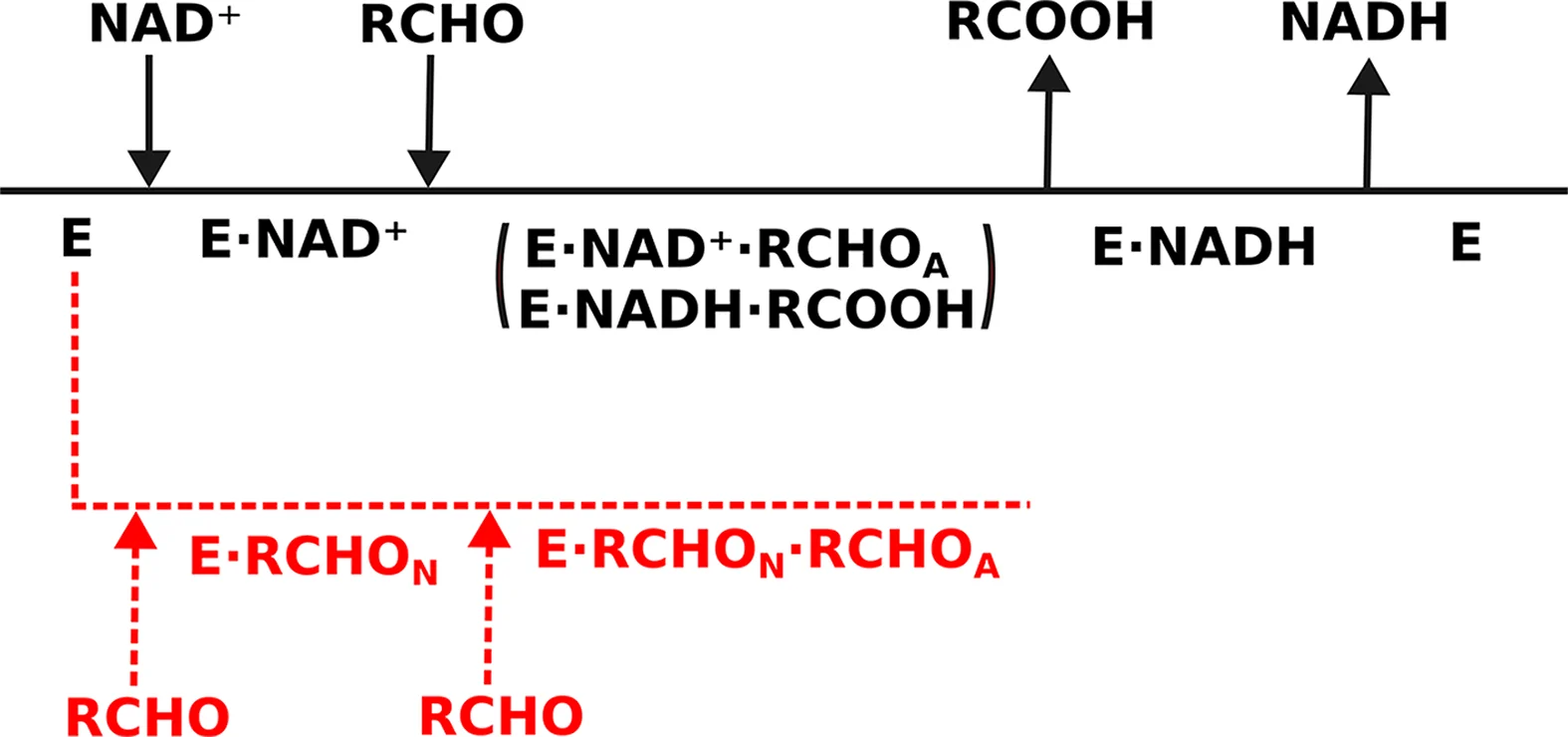
Proposed model for substrate inhibition of PA2125 by 3-OHBZAL and 3,5-diOHBZAL In the catalytic pathway (solid black lines), NAD^+^ binds first at the nucleotide-binding site, followed by aldehyde binding at the aldehyde-binding site. In the inhibitory pathway (red dashed lines), aldehyde binding at the nucleotide-binding site forms a nonproductive binary dead-end complex, followed by binding of a second aldehyde molecule at the aldehyde-binding site to form a nonproductive ternary dead-end complex. RCHO_A_ and RCHO_N_ denote aldehyde bound at the aldehyde- and nucleotide-binding sites, respectively. The scheme follows the Cleland convention [16]; the unidirectional arrows indicate the order of substrate or inhibitor addition and product release but do not imply irreversible steps.

In an ordered sequential steady-state Bi Bi mechanism such as that exhibited by PA2125, for a total competitive substrate-inhibition model in which the aldehyde (substrate B in Cleland notation [16]) competes with NAD^+^ (substrate A) for binding to the free enzyme, the inhibitory factor (1 + [B]/*K*_IS_) would be expected to affect both the constant term (*K*_iA_*K*_B_), accounting for formation of an inhibitory binary E·B complex, and the *K*_A_[B] term, accounting for formation of a nonproductive ternary complex containing two aldehyde molecules (E·B·B) [17]. However, the best fits were obtained with a reduced equation in which the inhibitory term affected only *K*_A_[B]. Thus, the kinetically relevant inhibitory species appears to be a catalytically incompetent enzyme species involving two aldehyde molecules, whereas the binary E·B complex, if formed, does not accumulate detectably under steady-state conditions. One possible explanation is that binding of one aldehyde molecule to the nicotinamide nucleotide-binding site induces binding of a second aldehyde molecule to the aldehyde-binding site in a highly cooperative manner, thereby forming the abortive, dead-end E·RCHO_N_·RCHO_A_ complex, where E·RCHO_N_ and E·R-CHO_A_ denote aldehyde molecules bound to the nucleotide- and aldehyde-binding sites, respectively (Figure 5).

To further test the hypothesis that substrate inhibition in PA2125 involves competition between the inhibitory aldehyde molecule and NAD^+^ for the nucleotide-binding site, we examined the effect of exogenous NADH on substrate inhibition by 3-OHBZAL and 3,5-diOHBZAL (Figure 4C,D and Table 3). Aldehyde-saturation curves were obtained at a fixed NAD^+^ subsaturating concentration and in the absence and presence of 80 μM NADH, and each curve was fitted independently to the substrate-inhibition equation (eqn 3). *V*_max_,_app_ decreases in the presence of NADH, consistent with product inhibition, whereas *K*_IS,app_ increased (insets to Figure 4C,D). Thus, although exogenous NADH decreased PA2125 activity through product inhibition, it simultaneously attenuated the observable substrate inhibition by the aldehyde.

A similar nucleotide-competitive substrate-inhibition mechanism has also been reported for an ALDH of human erythrocytes [18], the nonphosphorylating glyceraldehyde-3-phosphate dehydrogenase from *Streptococcus mutans* [19], and the succinic semialdehyde dehydrogenase from *Streptococcus pyogenes* [20]. In the latter case, this inhibition mechanism was supported by the crystal structure of an inhibitory complex. In the absence of a PA2125 crystal structure, we performed docking simulations using a structural model generated with AlphaFold to assess whether binding of GSA, 3-OHBZAL, and 3,5-diOHBZAL to the nicotinamide nucleotide-binding site was structurally plausible. In these docking poses, the three aldehydes interacted with residues located near the region that accommodates the nicotinamide ribose moiety of the nucleotide (Supplementary Figure S5). Notably, this region includes Glu383 (PA2125 numbering, Glu399 in human ALDH2), a glutamate that interacts with the nicotinamide ribose of NAD^+^ [21] and one of the earliest recognised strictly conserved residues of the ALDH superfamily [22]. In addition, two other residues contacted by the aldehydes, Trp148 and Arg332, were also predicted to interact with NAD^+^ in the nucleotide-docking model. These shared interactions support the hypothesis that aldehyde binding at this position could interfere with productive NAD^+^/NADH binding. Interestingly, in preliminary blind-docking trials performed in the absence of NAD^+^, the aldehydes were frequently predicted to occupy the nucleotide-binding cavity rather than the aldehyde-binding pocket adjacent to Cys282. Docking in the NAD^+^-bound enzyme constrained aldehyde binding to the catalytically relevant pocket (see below), consistent with the ordered kinetic mechanism inferred from the initial-velocity patterns.

Although the docking results suggested that GSA, 3-OHBZAL, and 3,5-diOHBZAL can all be accommodated within the nicotinamide nucleotide-binding cavity, only the latter two aldehydes showed strong substrate inhibition. This apparent discrepancy can be explained by considering that docking identifies structurally plausible binding modes but does not determine whether the corresponding complexes accumulate under steady-state conditions sufficiently to affect the observed kinetics or compete effectively with the productive catalytic route. In the case of GSA, the very high *k*_cat_, low *K*_B_, and consequently very high *k*_cat_/*K*_B_, indicate that formation and turnover of the productive enzyme·NAD^+^·GSA complex are strongly favoured. By contrast, for 3-OHBZAL and 3,5-diOHBZAL, the balance between productive oxidation and nonproductive aldehyde binding appears to shift toward formation of inhibitory species even at low aldehyde concentration. Thus, substrate inhibition does not depend solely on the ability of an aldehyde to bind at this site, but on whether such binding is kinetically competitive with the productive catalytic route under steady-state conditions.

### Docking of the hydroxybenzaldehydes GSA, 3-OHBZAL, and 3,5-diOHBZAL in the PA2125 aldehyde-binding site and molecular dynamics

To investigate the structural basis of substrate recognition by PA2125, GSA, 3-OHBZAL, and 3,5-diOHBZAL, they were docked into the catalytic aldehyde-binding site using a model of the PA2125·NAD^+^ complex as described in the ‘Materials and methods’ section. The resulting PA2125·NAD^+^·aldehyde models are consistent with the ordered sequential steady-state Bi Bi mechanism inferred from the kinetic data. The inclusion of NAD^+^ in the model directed the analysis toward productive aldehyde binding in the catalytic site by occupying the nucleotide-binding cavity, and the docking box was centred on Cys282 to restrict the search to poses compatible with nucleophilic attack by the catalytic cysteine.

**Figure 6:**
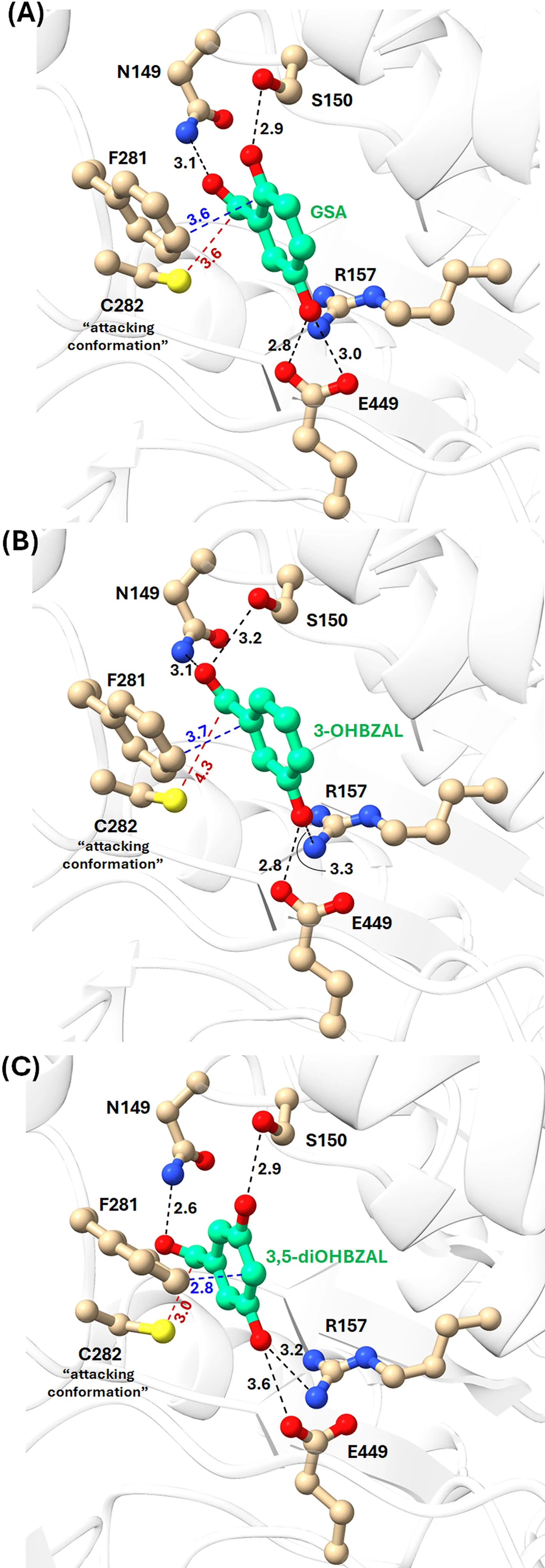
Proposed binding modes of GSA, 3-OHBZAL, and 3,5-diOHBZAL in the active site of PA2125 Close-up views of modelled PA2125·NAD^+^ complexes with GSA (**A**), 3-OHBZAL (**B**), and 3,5-diOHBZAL (**C**). Selected active-site residues are shown as sticks and the aldehydes as ball-and-stick models. Dashed lines indicate predicted polar contacts or short intermolecular distances; values are given in Å. The aldehyde carbonyl carbon is positioned near the Sγ atom of catalytic Cys282.

The docking poses were analysed with particular attention to the position of the aldehyde carbonyl relative to the Sγ of the catalytic cysteine (Cys282) and to interactions that could stabilise productive binding. This analysis identified Ser150, Arg157, Phe281, and Glu449 as putative determinants of aldehyde binding and positioning in PA2125 (Figure 6). These residues are noteworthy because they represent unusual amino acid substitutions at positions that are generally conserved among ALDHs. Positions equivalent to Ser150, Arg157, and Glu449 are frequently aromatic in many ALDHs, whereas the position corresponding to Phe281 is typically non-aromatic. Within the ALDH33A subfamily (to which PA2125 belongs, see below), Arg157, Phe281, and Glu449 are invariant in our alignment, whereas Ser150 is replaced by alanine in a small number of sequences, consistent with the potential role for these positions in substrate recognition suggested by docking simulations. Their individual contributions to substrate binding and catalysis are currently being investigated by site-directed mutagenesis.

Because docking provides static poses and approximate binding scores and is subject to recognised methodological limitations [23,24], selected docking-derived complexes were subjected to MD simulations to determine whether the aldehydes remained accommodated in the active-site region and sampled geometries compatible with nucleophilic attack by Cys282. As the simulations were initiated from poses in the catalytic aldehyde-binding site, they were interpreted primarily in terms of productive substrate accommodation and catalytic positioning, rather than nonproductive aldehyde binding in the nucleotide-binding cavity.

Root-mean-square deviation (RMSD) analysis of ligand heavy atoms showed that the three aldehydes remained associated with the active-site region but differed in their conformational heterogeneity (Supplementary Figure S6). In the GSA complex, two trajectories remained at comparatively lower ligand RMSD values, whereas one replicate shifted to a higher-RMSD state, indicating that the substrate can sample alternative conformations while remaining associated with the active-site region. The 3-OHBZAL and 3,5-diOHBZAL complexes showed greater variability among trajectories, with several replicates sampling more displaced ligand conformations. Thus, although RMSD analysis did not reveal a single behaviour for each ligand, 3-OHBZAL and 3,5-diOHBZAL showed greater conformational heterogeneity than GSA, in qualitative agreement with their poorer catalytic performance.

**Figure 7:**
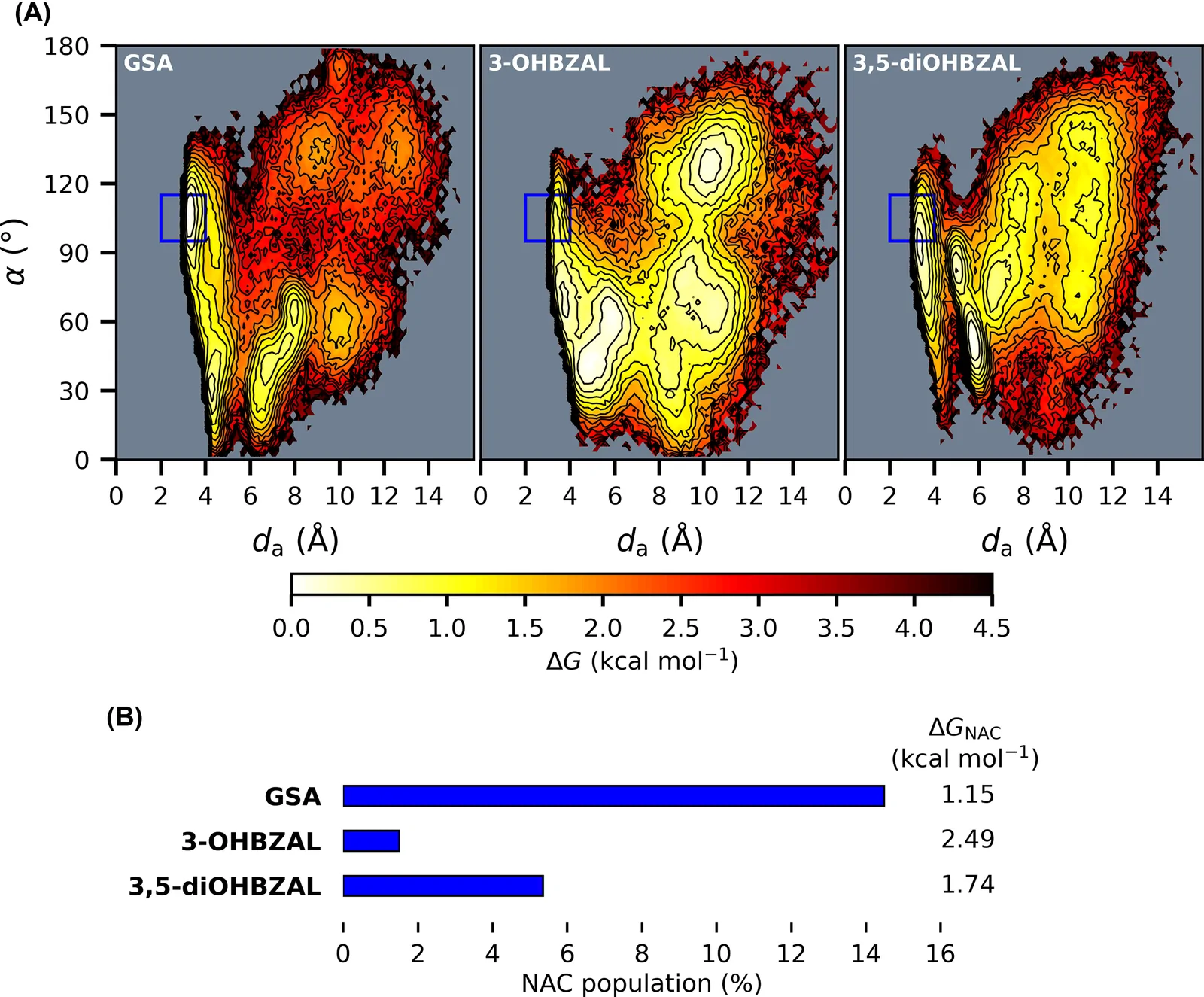
MD-derived free-energy landscapes and NACs of PA2125 complexes with GSA, 3-OHBZAL, and 3,5-diOHBZAL (**A**) Population-derived free-energy maps generated from the pooled trajectories of three independent MD replicates for each PA2125·NAD^+^·aldehyde complex, using the Cys282 Sγ-aldehyde carbon distance (*d*_a_) and Bürgi–Dunitz angle (α) as geometric descriptors. Lower Δ*G* values denote more highly populated conformations, and unsampled regions are shown in grey. The blue box indicates the NAC region. (**B**) NAC populations calculated from the pooled trajectories; the corresponding Δ*G*_NAC_ values are shown at the right.

To determine whether the kinetic differences were related to productive substrate positioning, near-attack conformations (NACs) were quantified from the Cys282 Sγ-aldehyde carbon distance, *d*_a_, and the Bürgi–Dunitz angle, α, using the criteria defined in the ‘Materials and methods’ section. Two-dimensional free-energy maps were generated from the pooled trajectories of the three independent replicas for each aldehyde (Figure 7A), and NAC populations and population-derived Δ*G*_NAC_ values were calculated for direct comparison among substrates (Figure 7B).

In all three PA2125·NAD^+^·aldehyde complexes, the aldehyde sampled multiple geometrical states during the simulations rather than remaining in a single stable reactive pose. However, the ability to populate the NAC region differed markedly among substrates. GSA showed the highest NAC population, with 14.5% of the frames falling within the defined NAC region, corresponding to a population-derived Δ*G*_NAC_ of 1.15 kcal mol^-1^. In contrast, 3-OHBZAL showed the lowest NAC population, with only 1.5% of the frames in the NAC region, and the highest Δ*G*_NAC_ value, 2.5 kcal mol^-1^. 3,5-DiOHBZAL displayed intermediate behaviour, with a NAC population of 5.35% and a Δ*G*_NAC_ of 1.74 kcal mol^-1^. Thus, the order of NAC population was GSA > 3,5-diOHBZAL > 3-OHBZAL.

As a complementary analysis, transition networks were constructed from the same *d*_a_,α space, which was partitioned into states, to compare accessibility to the NAC region among the three aldehyde complexes (Supplementary Figure S7). In all cases, NAC states were sampled, indicating that the aldehydes can adopt geometries compatible with nucleophilic attack by Cys282. However, the connectivity of the non-NAC states to the NAC region differed among substrates. In the GSA complex, several non-NAC states were readily connected to NAC states, as indicated by relatively high one-step probabilities of reaching NAC and by mean first-passage time (MFPT) values mostly below 300 frames. The 3-OHBZAL complex showed a similar overall organisation, but a larger fraction of states required longer first-passage times to reach NAC. This reduced connectivity was most pronounced for 3,5-diOHBZAL, for which many populated states showed very low one-step probabilities of reaching NAC and MFPT values extending to approximately 500 frames. Thus, NAC occupancy and NAC accessibility did not rank the substrates in the same way: 3,5-diOHBZAL showed a higher overall NAC population, whereas 3-OHBZAL appeared to maintain more favourable connectivity between non-NAC and NAC states. These opposing tendencies may partially offset each other, consistent with the similar apparent catalytic behaviour of the two aldehydes.

To assess whether differences in overall binding energetics could account for the kinetic behaviour of PA2125 for GSA, 3-OHBZAL, and 3,5-diOHBZAL, the binding energy (Δ*G*_bind_) of each aldehyde to the PA2125·NAD^+^ complex, calculated considering only NAC conformations, was estimated using the MM-PBSA method. The estimated values were −16.2 ± 2.8, −12.6 ± 2.9, and −18.7 ± 2.2 kcal mol^−1^ for GSA, 3-OHBZAL, and 3,5-diOHBZAL, respectively. Given the approximations inherent to the MM-PBSA method [25], these values were interpreted as comparative binding-energy estimates rather than as absolute binding free energies. These favourable binding free-energy estimates support the ability of the PA2125 active site to accommodate all three aldehydes but do not fully explain the differences in their catalytic efficiency. Instead, the *d*_a_,α-based descriptors, which more directly reflect pre-reactive positioning, suggest that catalytic efficiency depends less on the ability of the aldehyde to bind into the catalytic pocket than on its capacity to sample and recover catalytically competent geometries.

Taken together, the docking and MD analyses provide integrated structural and dynamic insights into substrate recognition and catalytic positioning in PA2125. All three aldehydes remained accommodated in the catalytic aldehyde-binding site, in qualitative agreement with their similar *K*_M_ values. However, GSA more frequently populated and more readily reached, geometries compatible with nucleophilic attack by Cys282. This provides a plausible structural-dynamic rationale for the higher *k*_cat_ observed with GSA, which largely accounts for its higher *k*_cat_/*K*_M_ given the similar *K*_M_ values of the three aldehydes. Because these analyses are based on classical MD simulations and operational geometric definitions of pre-reactive states, they should be interpreted as qualitative evidence of differences in conformational sampling rather than as direct simulations of the chemical step.

### Possible biological and biotechnological implications of PA2125 substrate specificity

Beyond their mechanistic interest, the preference of PA2125 for selected OHBZALs suggests a role in the oxidation of phenolic aldehyde intermediates formed during the intracellular degradation of plant-derived or anthropogenic aromatic compounds encountered by *P. aeruginosa*. Oxidation of the methyl group of substituted toluenes can generate the corresponding substituted benzaldehydes as pathway intermediates. Hydroxytoluenes such as m-cresol, toluquinol, and orcinol may arise through bacterial transformation of lignin or fungal secondary metabolism in plant-associated niches and could therefore represent environmental precursors of the corresponding OHBZALs, 3-OHBZAL, and GSA [26–29].

The activity of PA2125 with chloro- and nitroBZALs further broadens its substrate scope and may be relevant to the biodegradation of chlorotoluenes and nitrotoluenes. Chlorotoluenes are mainly anthropogenic compounds produced industrially by chlorination of toluene and used as chemical intermediates or solvents. In bacteria possessing benzylic side-chain oxidation pathways, some chlorotoluene isomers can be converted into the corresponding chloroBZALs [30], although such pathways have not been demonstrated in *P. aeruginosa*. Similarly, the bacterial degradation of 4-nitrotoluene has been shown to proceed through successive oxidation of the methyl group to the corresponding alcohol, aldehyde, and carboxylic acid [31], illustrating that nitroBZALs can arise as intracellular intermediates during the catabolism of nitroaromatic compounds. From a biotechnological perspective, the ability of PA2125 to oxidise chloro- and nitroBZALs is relevant to bioremediation applications. Chlorotoluenes and nitrotoluenes are persistent environmental contaminants, and their biodegradation requires enzymatic activities capable of processing the aldehyde intermediates generated during side-chain oxidation. The broad acceptance of halogenated and nitro-substituted BZALs by PA2125 suggests that this enzyme could contribute to the detoxification of such intermediates in bioremediation contexts, either in native *P. aeruginosa* strains or as a component of engineered biodegradation pathways.

Another class of aromatic aldehydes oxidised by PA2125 are pyridine carbaldehydes. Pseudomonads are known to metabolise pyridine-containing compounds, including intermediates of nicotine degradation, and bacterial enzymes acting on pyridine-containing aldehyde intermediates have been described in such pathways [32,33]. However, the pyridinecarbaldehydes tested here have not been identified as intermediates in any defined metabolic pathway in *P. aeruginosa* PAO1; they are therefore best regarded as probe substrates that help define the catalytic scope of PA2125 rather than as likely physiological substrates.

### Comparative sequence and phylogenetic analyses identify PA2125 as member of a novel ALDH family

**Figure 8:**
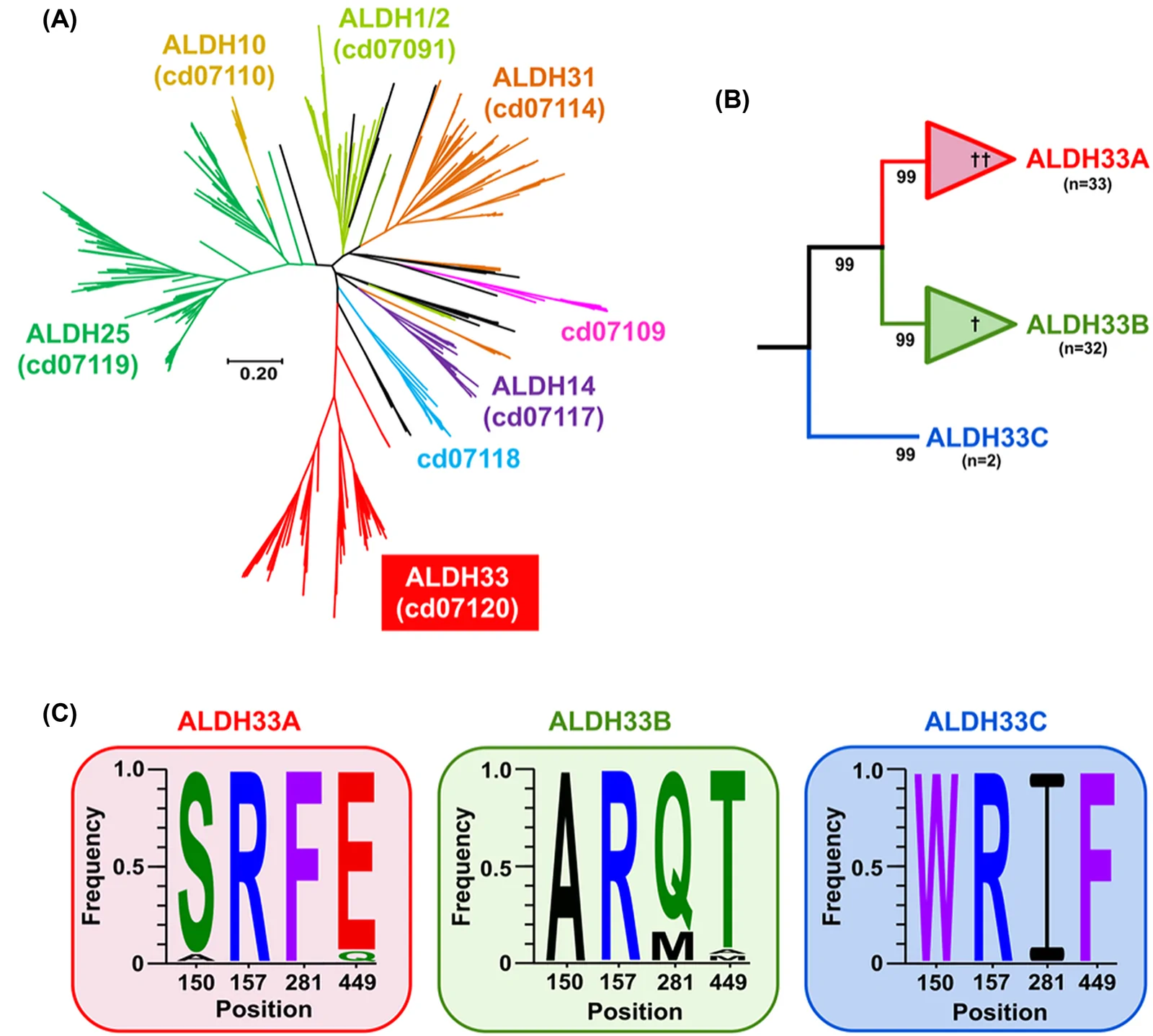
Phylogenetic and residue-conservation analyses of PA2125-related ALDH33 proteins (**A**) Unrooted maximum-likelihood tree of ALDH sequences retrieved using PA2125 as query. Established ALDH family designations are indicated, and the cd07120 clade, here designated ALDH33, is highlighted in red. Branch lengths are proportional to the number of amino acid substitutions per site; the scale bar represents 0.20 substitutions per site. (**B**) Schematic representation of the rooted maximum-likelihood tree of ALDH33 proteins. ALDH33A, ALDH33B, and ALDH33C subfamilies are shown as collapsed clades comprising 33, 32, and 2 sequences, respectively. Bootstrap support values are shown adjacent to the corresponding nodes. Daggers indicate the sequences discussed in the text: PA2125 and the previously reported gentisaldehyde dehydrogenase in ALDH33A, and the proposed furfural dehydrogenase in ALDH33B. The complete tree and sequence information are provided in Supplementary Figure S8 and Supplementary Table S3, respectively. (**C**) Sequence logos showing residue conservation at positions 150, 157, 281, and 449 (PA2125 numbering) in the three ALDH33 subfamilies. Amino acids are coloured according to side-chain properties: hydrophobic/aliphatic, black; polar uncharged, green; positively charged, blue; negatively charged, red; and aromatic, purple.

A phylogenetic analysis of 481 ALDH protein sequences sampled from a set of 3559 non-redundant prokaryotic genomes (representing 15 345 complete genomes) showed that PA2125 clusters within a distinct, well-supported clade (Figure 8). This clade corresponds to the NCBI Conserved Domain Database [34] conserved domain family cd07120 and is here designated the ALDH33 family, following the nomenclature system for the ALDH gene superfamily based on divergent evolution and sequence identity thresholds [35], which at the time comprised 24 families (ALDH1 to ALDH24) [36]. Families ALDH25, ALDH26, and ALDH27 were established in earlier work from our group [37], and families ALDH28 to ALDH32 are described in a manuscript currently in preparation by our group. Notably, most of the 481 sequences belonged to ALDH families other than cd07120, indicating that the BLASTP search also recovered related ALDH families, but all 67 cd07120 sequences were recovered using both the less stringent E-value threshold of 1 × 10^−60^ and the more stringent threshold of 1 × 10^−90^. The less stringent threshold increased the number of homologues belonging to other ALDH families but did not recover any additional cd07120 members. Thus, the more stringent threshold did not exclude any sequence of the ALDH33 family present in the surveyed genome set.

A targeted phylogenetic analysis of the ALDH33 family (*n* = 67 sequences) was then performed, and the best-scoring maximum-likelihood tree (log-likelihood = −180 997.09) is shown as a collapsed representation in Figure 8B and as the complete tree in Supplementary Figure S8, and full sequence and taxonomic information is provided in Supplementary Table S3. The analysis suggests three subfamilies within the ALDH33 family: subfamily A, which includes PA2125 and an ALDH encoded by *nolF* in *Burkholderia* sp. strain BC1 (69% identity to PA2125) that was proposed to act as a gentisaldehyde dehydrogenase based on activity detected in crude extracts of cells grown on 2-hydroxy-1-naphthoic acid [8]; subfamily B, which includes an ALDH encoded by *psfA* in *Pseudomonas putida* (53% identity to PA2125) that has been proposed to function as a furfural dehydrogenase because *psfA* expression was induced by furfuryl alcohol, furfural, or furoic acid; and subfamily C, which is represented in this tree by two Bacillota sequences. Additional closely related Bacillota homologues were identified by BLASTP, which suggests that ALDH33C is not limited to the sequences sampled in the tree. However, because no member of this proposed subfamily has been experimentally characterised, its taxonomic distribution, substrate range, and physiological role remain to be established.

Notably, the three ALDH33 subfamilies show changes at positions 150, 281, and 449 (PA2125 numbering) (Figure 8C). By contrast, Arg157 was conserved across these groups, as were the catalytic residues Cys282, Asn149, and Glu248 (not shown). This conservation pattern suggests that, although the catalytic machinery is preserved across the ALDH33 family, variation in other residues lining the aldehyde-binding site may contribute to subfamily-specific substrate preferences. This interpretation is consistent with our finding that PA2125 shows negligible activity with furfural under our assay conditions, whereas the *P. putida* PsfA enzyme, which belongs to the ALDH33B subfamily, has been associated with furfural-related metabolism.

Within the dataset studied, ALDH33 homologues were found in a restricted but taxonomically diverse subset of bacteria. In our survey, most sequences belonged to Pseudomonadota (formerly Proteobacteria), whereas Actinomycetota formed a substantial minority; Cyanobacteriota and Bacteroidota were represented by single sequences, and Bacillota by only two sequences (Supplementary Table S3). This distribution suggests that, at least within the sequence space sampled here, ALDH33 is not a ubiquitous ALDH family, but rather a specialised bacterial lineage that may be associated with particular metabolic contexts, possibly including the metabolism of aromatic compounds.

Overall, the ALDH33 family remains poorly characterised, and its taxonomic distribution and functional diversity may be broader than suggested by the representative sequence set analysed here. Thus, the biochemical and structural characterisation of PA2125 provides a starting point for understanding substrate specificity and functional diversification within this emerging ALDH family.

### Integrated view of PA2125 substrate specificity and functional assignment

Taken together, the kinetic and computational results identify PA2125 as an ALDH with a marked preference for selected OHBZALs. GSA is distinguished by its very high catalytic efficiency and lack of detectable substrate inhibition, whereas the MD analyses suggest that its superior catalytic performance is associated with more frequent and more readily accessible pre-reactive geometries. These findings support the functional assignment of PA2125 as a gentisaldehyde dehydrogenase and suggest that GSA is its most efficient physiological substrate. However, 3-OHBZAL and 3,5-diOHBZAL are also efficiently oxidised and may represent additional physiological substrates, given that the corresponding hydroxytoluenes can be encountered by *P. aeruginosa* in its environment. Regarding 2,4,5-triOHBZAL, the precise metabolic role in *P. aeruginosa* remains to be established. Studies aimed at defining the physiological role of PA2125 and its integration into an aromatic compound degradation pathway are currently under way.

## Materials and methods

### Chemicals and biochemicals

NAD^+^ (Sigma–Aldrich, cat. no. N7004, ≥96.5% purity), NADP^+^ (Sigma–Aldrich, cat. no. N0505, ≥98% purity), NADH (Sigma–Aldrich, cat. no. N8129, ≥97% purity), TCEP, DTT, β-mercaptoethanol, and the tested aldehydes were obtained from Sigma–Aldrich Química, S.A. de C.V. (Toluca, México), except 2,6-diOH-BZAL, 2,4,5-triOH-BZAL, and 4-isopropilBZAL, which were purchased from Santa Cruz Biotechnology (Inc., Dallas, TX, U.S.A.). The free forms of the diethylacetal aldehydes were prepared by acid hydrolysis as described [12] and immediately used or stored at −70°C until use.

### Cloning of the *PA2125* gene

*Pseudomonas aeruginosa* genomic DNA was prepared as described by Ausubel et al. [38] and used as template for polymerase chain reaction (PCR) amplification using the primers 5′-GGAATTCCATATGAACGGACACGCCAGACACTGG-3′ (forward) and 5′-CCCAAGCTTTCAGGCCGCGCCGGTGGTCAG-3′ (reverse), designed based on the PA2125 sequence retrieved from the *P. aeruginosa* genomic database (https://www.pseudomonas.com/). The PCR product was purified using a GeneJET PCR Purification Kit (Thermo Scientific, Waltham, MA, U.S.A.) and cloned into the pET-28b(+) vector using the NdeI and HindIII restriction sites. The resulting plasmid, pET-28b(+)-*PA2125*, containing the full *PA2125* coding sequence fused to an N-terminal His_6_ tag, was used to transform competent *Escherichia coli* XL10-Gold cells (Stratagene, La Jolla, CA, U.S.A.). Plasmid DNA was extracted from cells selected on kanamycin and purified using the GeneElute kit (Sigma–Aldrich, St. Louis, MO, U.S.A.). The insert was sequenced using the T7 primer (Laragen Inc., U.S.A.). After confirming that the PA2125 coding sequence was in frame and contained no mutations, the plasmid was used to transform competent *E. coli* Rosetta(DE3)pLysS cells (Novagen, Merck Millipore, Darmstadt, Germany) for protein overexpression. Transformed cells were stored as 30% (v/v) glycerol stocks at −70°C until use.

### Expression and purification of PA2125

Cells transformed with pET-28b(+)-*PA2125* were grown in Luria–Bertani broth supplemented with 0.1 mg ml^−1^ kanamycin to an OD_600_ of 0.5–0.6. Protein expression was induced with 0.1 mM isopropyl β-D-1-thiogalactopyranoside (IPTG) for 4 h at 37°C, and the cells were harvested by centrifugation at 5860 × ***g*** for 10 min. The cell pellet was resuspended in 50 mM potassium phosphate buffer, pH 6.9, containing 200 mM KCl, 20% (v/v) ethylene glycol, 1 mM EDTA, and 10 mM β-mercaptoethanol (Buffer A). Cells were lysed by sonication on ice for 25 min, and cell debris was removed by centrifugation at 26 900 × ***g*** for 20 min at 4°C. The supernatant was filtered and loaded onto a nickel-affinity column (Protino Ni-TED; Macherey-Nagel, Düren, Germany) equilibrated with Buffer A modified by omitting EDTA and reducing β-mercaptoethanol to 2 mM. The column was washed with the same buffer, and PA2125 was eluted with 150 mM imidazole in Buffer A. Imidazole was removed from fractions showing benzaldehyde dehydrogenase activity by desalting using a HiPrep 26/10 desalting column (Cytiva, Marlborough, MA, U.S.A.) coupled to an ÄKTA pure FPLC system (Cytiva). Enzyme aliquots were stored in Buffer A at −70°C until use.

Protein purity was assessed by SDS–PAGE according to Laemmli [39]. Protein concentration was determined spectrophotometrically at 280 nm using a molar absorptivity of 50,420 M^−1^ cm^−1^ for the His_6_-tagged PA2125 subunit, calculated from its amino acid sequence using ExPASy ProtParam [10]. The apparent native molecular mass of purified PA2125 was estimated by size-exclusion chromatography using a Superdex 200 column (Cytiva) equilibrated with 50 mM potassium phosphate buffer, pH 7.0, containing 50 mM KCl, 10% (v/v) glycerol, and 10 mM β-mercaptoethanol. PA2125 was loaded onto the column at a concentration of 1 mg ml^−1^. The column was calibrated using molecular-mass standards (Bio-Rad Laboratories, Inc., U.S.A.), and the apparent molecular mass was estimated from the relationship between log(molecular mass) and elution volume [40].

### Activity assays and kinetic characterisation

Activity assays were performed at 30°C in a final volume of 0.5 ml containing 100 mM potassium phosphate buffer (pH 8.0) and the concentrations of substrates and products stated in each experiment. Reactions were initiated by addition of 40 nM PA2125 (final monomer concentration) except for assays with GSA, 3-OHBZAL, and 3,5-diOHBZAL, in which 2 nM enzyme (final monomer concentration) was used. NAD(P)H formation was monitored spectrophotometrically in 1.0 cm path-length cuvettes at 340 nm (ε_340_(NAD(P)H) = 6220 M^−1^ cm^−1^ [41]). NAD^+^ concentration was determined spectrophotometrically at 260 nm using ε_260_ = 18 000 M^−1^ cm^−1^ [42]. The exact concentration of aldehyde stock solutions was determined in each experiment by quantifying the amount of NADH formed upon complete oxidation of the aldehyde in the PA2125-catalysed reaction in the presence of an excess of NAD^+^. For substrates and their corresponding products exhibiting absorbance at 340 nm, molar absorptivities were determined under the assay conditions, and the effective molar absorptivity used to calculate the enzyme activity with this substrate was corrected as:

(1) $$\varepsilon_{340,\;\text{corr}}=\varepsilon_{340\;(\text{NADH})}+\varepsilon_{340\;(\text{R}-\text{COOH})}-\varepsilon_{340\;(\text{R}-\text{CHO})}$$

where ε_340_,_corr_ is the corrected molar absorptivity, ε_340 (NADH)_ is the molar absorptivity of NADH, and ε_340(R-COOH)_ and ε_340(R-CHO)_ are the molar absorptivities of the carboxylic acid product and the corresponding aldehyde substrate, respectively. The ε_340 (R-COOH)_ and ε_340(R-CHO)_ values used for these corrections are provided in Supplementary Table S1.

Initial velocities were determined from the initial linear portions of reaction progress curves. For each substrate concentration, initial velocities were determined in duplicate or triplicate and averaged; complete saturation curves were independently repeated at least twice (typically three times), using different enzyme preparations. Enzyme activity was expressed as specific activity (U mg^−1^ prot.), where one unit of activity (U) is defined as the amount of enzyme that produces 1 μmol of NADH per min under the assay conditions. Turnover numbers were expressed per active site, using the enzyme concentration calculated from the theoretical molecular mass of the PA2125 subunit.

Kinetic parameters were estimated by nonlinear regression fitting of the initial-velocity data with GraphPad Prism (version 11.0.2 for Windows, GraphPad Software, Boston, MA, U.S.A.). Individual fits were made to the Michaelis-Menten equation (eqn 2) or to the equation describing total substrate inhibition (eqn 3):

(2) $$v_{0}=\frac{V_{\text{max}}[\text{S}]}{{\text{K}}_{\text{M}}+[\text{S}]}$$

(3) $$v_{0}=\frac{V_{\text{max}}[\text{S}]}{{\text{K}}_{\text{M}}+[\text{S}](1+[\text{S}]/{\text{K}}_{\text{IS}})}$$

where *v*_0_ is the measured initial velocity; *V*_max_ is the maximal velocity; [S] is the variable substrate concentration; *K*_M_, Michaelis–Menten constant, is the substrate concentration at half-maximal velocity in the absence of inhibition; and *K*_IS_ is the inhibition constant of the aldehyde.

Inhibition by 4-OHBZAL was analysed by fitting initial velocities to (eqn 4):

(4) $$v_{\text{i}}=\frac{(v_{0}-v_{\infty })\;I_{50}}{I_{50}+[\text{I}]}+v_{\infty }$$

where *v*_i_ and *v*_0_ are experimental initial velocities measured in the presence and absence of inhibitor, respectively; *v*_∞_ is the residual velocity at saturating inhibitor concentration; [I] is the inhibitor concentration; and *I*_50_ is the estimated 4-OHBZAL concentration at which *v*_i_ = (*v*_0_ + *v*_∞_)/2 under the assay conditions used.

The kinetic mechanism for GSA, 3-OHBZAL, and 3,5-diOHBZAL was identified by analysing initial-velocity and NADH-inhibition patterns. The corresponding data were globally fitted to the initial-velocity equation for an ordered sequential steady-state Bi Bi mechanism in the absence (GSA) or presence of total substrate inhibition (3-OHBZAL and 3,5-diOHBZAL) (eqns 5 and 6, respectively), or to (eqns 7 and 8) corresponding to competitive or mixed inhibition by NADH, as appropriate:

(5) $$v_{0}=\frac{V[\text{A}][\text{B}]}{K_{\text{iA}}K_{\text{B}}+K_{\text{B}}[\text{A}]+K_{\text{A}}[\text{B}]+[\text{A}][\text{B}]}$$

(6) $$v_{0}=\frac{V[\text{A}][\text{B}]}{K_{\text{iA}}K_{\text{B}}+K_{\text{B}}[A]+K_{\text{A}}[\text{B}](1+[\text{B}]/K_{\text{IS}})+[\text{A}][\text{B}]}$$

(7) $$v_{0}=\frac{V[\text{S}]}{K_{\text{M}}(1+[\text{I}]/K_{\text{is}})+[\text{S}]}$$

(8) $$v_{0}=\frac{V[\text{S}]}{K_{\text{M}}(1+[\text{I}]/K_{\text{is}})+[\text{S}](1+[\text{I}]/K_{\text{ii}})}$$

where A and B are the first and second substrates, respectively; *K*_A_ and *K*_B_ are the Michaelis–Menten constants for substrates A and B, respectively; *K*_iA_ is the dissociation constant of the complex of the enzyme with A; *K*_IS_ is the inhibition constant of substrate B; and *K*_is_ and *K*_ii_ are the slope and intercept inhibition constants for NADH (Cleland nomenclature), corresponding to competitive and uncompetitive inhibition, respectively. In all cases, standard errors for the estimated *k*_cat_/*K*_M_ values were calculated by propagation of uncertainty, including the covariance between the estimated *k*_cat_ and *K*_M_ values. The choice between the model with *K*_i__A_ fixed to *K*_A_ and the model with *K*_i__A_ and *K*_A_ estimated independently was based on an extra-sum-of-squares F-test for nested models, performed in GraphPad Prism.

### Theoretical p*K*_a_ values calculation

The p*K*_a_ values and microspecies distribution of 4-OHBZAL and 2,4,5-triOHBZAL at pH 8.0 were predicted using Chemicalize (ChemAxon, Budapest, Hungary). The lowest acidic p*K*_a_ was assigned to the C4-OH group.

### Molecular docking of the aldehyde substrates and MD simulations

A three-dimensional monomeric model of PA2125 was generated with AlphaFold2 [43]. NAD^+^ was positioned in the nucleotide-binding site by structural superposition followed by coordinate transfer from the NAD^+^-bound betaine aldehyde dehydrogenase BetB P449M/Y450L double mutant from *Staphylococcus aureus* (PDB: 5EZ4 [44]). The resulting PA2125·NAD^+^ model was prepared with CHARMM-GUI and energy-minimised in CHARMM v47 before docking. Ligand structures for GSA, 3,5-diOHBZAL, 3-OHBZAL, and 4-OHBZAL were obtained from PubChem (CIDs 70 949, 94 365, 101, and 126, respectively [45]), built and energy-minimised in Avogadro 1.2.0 using the MMFF94 force field [46]. The phenolic hydroxyl groups of the ligands were modelled in their protonated states, except for 4-OHBZAL, which was modelled in its deprotonated form to represent the predominant species at the assay pH of 8.0, because of its reported p*K*_a_ of 7.61 at 25°C [47]. Molecular docking was performed with AutoDock Vina v1.2.0 [48,49] using the Vina plugin in UCSF Chimera [50,51]. Docking was carried out on the PA2125·NAD^+^ complex, with NAD^+^ retained in the cofactor-binding site. The cubic search box was centred on the catalytic Cys282 Sγ atom and defined to include the catalytic pocket, using dimensions of 10 × 10 × 10 Å and grid-centre coordinates of x = −3.71, y = −2.40, and z = 1.34 Å. Vina was run with exhaustiveness = 8, num_modes = 10, and energy_range = 3 kcal mol^−1^. Docking poses were ranked by Vina score and inspected visually. Poses were selected for MD simulations based on catalytic plausibility, as assessed by three criteria: (i) proximity of the aldehyde carbonyl group to Cys282 Sγ, (ii) orientation of the carbonyl oxygen toward the putative oxyanion-stabilising region, and (iii) presence of plausible interactions with active-site residues.

Selected PA2125·NAD^+^·aldehyde complexes were used as starting structures for MD simulations. System building, ligand and cofactor parametrisation, solvation, and ion placement were performed using the Solution Builder workflow in CHARMM-GUI [52,53], which generated GROMACS-compatible topology and input files. Complexes were solvated with explicit TIP3P water in cubic boxes extending 15 Å beyond the protein surface, neutralised, and adjusted to 150 mM NaCl with Na^+^ and Cl^−^ ions. Simulations used the CHARMM36m force field [54]. Hydrogen mass repartitioning was applied to all hydrogen-bearing bonds to allow a 4-fs integration time step. The solvated systems were energy-minimised for 5000 steps, followed by 1 ns of solvent equilibration in the NVT ensemble and 1 ns of unrestrained MD in the NPT ensemble. Temperature was maintained at 300 K with the Nosé–Hoover thermostat, and pressure was maintained at 1 atm with the Parrinello–Rahman barostat. Van der Waals interactions were treated with a 12 Å cutoff using a force-switch modifier, and long-range electrostatic interactions were calculated using the particle-mesh Ewald method. Bonds involving hydrogen atoms were constrained with LINCS. Production simulations were performed in GROMACS v2025.3 [55] as three independent 100-ns replicas for each PA2125·NAD^+^·aldehyde complex, initiated with different random velocity seeds, yielding 300 ns of aggregate simulation time per complex.

Trajectory analysis was performed with built-in GROMACS tools and in-house Python scripts. Ligand heavy-atom RMSD values were calculated after alignment of the protein Cα atoms to the initial pose. To evaluate productive catalytic positioning, each MD frame was analysed using two geometric descriptors: the distance (*d*_a_) between the Cys282 Sγ atom and the aldehyde carbonyl carbon, and the attack angle α defined by Sγ-C=O at the carbonyl carbon, following the Bürgi–Dunitz convention [56,57]. NACs [58,59] were operationally defined as frames with *d*_a_ <4.0 Å and α between 95° and 115°. Two-dimensional free-energy maps were calculated from the sampled *d*_a_,α distributions, and relative free energies were calculated as Δ*G*(*d*_a_,α) = − *RT* ln[*P*(*d*_a_,α)/*P*_max_], where *P*(*d*_a_,α) is the probability of observing a given combination of *d*_a_ and α, *P*_max_ is the maximum probability observed for each aldehyde, *R* is the gas constant, and *T* = 300 K. The NAC population, *f*_NAC_, was calculated as the fraction of frames satisfying the NAC criteria, and ∆G_NAC_ was calculated as -*RT* ln(*f*_NAC_).

For transition-network analysis [60,61], the *d*_a_-α space was discretised into a two-dimensional grid of defined cell size (∆*d*_a_ = 0.2 Å, ∆α = 2.25°), and each populated grid cell was treated as a catalytic-geometry state. Transitions between states were obtained from consecutive frames across all three replicas. NAC accessibility was evaluated from the probability that a transition from a given state reached a NAC state in a single transition, and from the MFPT from each state to NAC, expressed in frames (1 frame = 1 ps). State free energies were calculated from state populations as *F_i_* = -*RT* ln(*P_i_*/*P*_max_), where *P_i_* is the occupancy probability of state *i* and *P*_max_ is the occupancy probability of the most populated state.

Binding-energy estimates for the aldehydes were calculated using the MM-PBSA method [62] as implemented in gmx_MMPBSA v1.6.4, based on MMPBSA.py v16.0 from AmberTools20 [63,64]. The PA2125·NAD^+^ complex was treated as the receptor and each aldehyde as the ligand. Calculations were performed using the single-trajectory approach, with solute and solvent dielectric constants of 1.0 and 78.5, respectively, and an ionic strength of 0.15 M. The nonpolar solvation contribution was estimated from the solvent-accessible surface area (SASA) using the default SASA-based nonpolar model (inp = 1) in gmx_MMPBSA. Entropic contributions were not included. The reported values represent the mean ± SD calculated from snapshots corresponding to NAC conformations sampled throughout the complete trajectory. To reduce temporal correlation between successive frames, only every 50th NAC-containing frame was included in the analysis, yielding a total of 869, 90, and 321 snapshots for GSA, 3-OHBZAL, and 3,5-diOHBZAL, respectively.

### Amino acid sequence analysis and phylogenetic studies

To reduce genomic redundancy, we used a set of 3559 representative prokaryotic genomes selected from 15 345 complete genomes by comparison and clustering based on the Genomic Similarity Score, as described [65]. PA2125 homologues were identified by BLASTP searches [66] against the NCBI RefSeq [67] protein records corresponding to these genomes. Sequences with E-values ≤1 × 10^−90^ were retained for the final dataset. Full-length sequences with adequate query coverage were aligned using MAFFT with the G-INS-i strategy [68]. Phylogenetic relationships were inferred using the maximum-likelihood method implemented in MEGA12 [69]. The best-fit substitution model was selected using the Model Selection tool in MEGA12 based on the Bayesian Information Criterion; the Le and Gascuel amino acid substitution model with empirical amino acid frequencies (LG + F) [70] was used. Rate variation among sites was modelled using a discrete gamma distribution with five categories (+G; shape parameter = 1.17), and 2.95% of sites were treated as evolutionarily invariant (+I). Branch support was assessed by bootstrap analysis [71] with 500 replicates. For the heuristic search, the initial tree was selected by comparing the log-likelihoods of a Neighbour-Joining (NJ) tree [72] and a Maximum Parsimony (MP) tree. The NJ tree was generated from a matrix of pairwise distances calculated using the LG + F model. The MP starting tree was the shortest tree obtained from 10 searches initiated from randomly generated trees. The final analysis included 481 protein sequences and 644 aligned positions. Sequence logos were generated using WebLogo3 [73].

## Supplementary Material

Supplementary Figure S1-S8 and Tables S1-S3

## Data Availability

The data supporting the findings of the present study are given in Figures 1–8, Tables 1–3, and in the supporting information for this article. Raw data are available on request.

## Open Access

Open access for this article was enabled by the participation of Universidad Nacional Autónoma de México in an all-inclusive Read & Publish agreement with Portland Press and the Biochemical Society.

## Abbreviations

ALDH: aldehyde dehydrogenase
BZAL: benzaldehyde
GSA: gentisaldehyde or 2,5-dihydroxybenzaldehyde
MD: molecular dynamics
MFPT: mean first-passage time
MP: Maximum Parsimony
NAC: near-attack conformations
NJ: Neighbour-Joining
PCR: polymerase chain reaction
PDB: Protein Data Bank
RMSD: root-mean-square deviation
SASA: solvent-accessible surface area
SDS–PAGE: sodium dodecyl sulfate–polyacrylamide gel electrophoresis

## References

[1] Feldman R.I. and Weiner H. (1972) Horse liver aldehyde dehydrogenase: II. Kinetics and mechanistic implications of the dehydrogenase and esterase activity. J. Biol. Chem. 247, 267–272 4336042 10.1016/S0021-9258(19)45785-2

[2] Cobessi D., Tête-Favier F., Marchal S., Branlant G. and Aubry A. (2000) Structural and biochemical investigations of the catalytic mechanism of an NADP-dependent aldehyde dehydrogenase from *Streptococcus mutans*. J. Mol. Biol. 300, 141–152 10864505 10.1006/jmbi.2000.3824

[3] Stover C.K., Pham X.Q., Erwin A.L., Mizoguchi S.D., Warrener P., Hickey M.J. et al. (2000) Complete genome sequence of *Pseudomonas aeruginosa* PAO1, an opportunistic pathogen. Nature 406, 959–964 10984043 10.1038/35023079

[4] Oberhardt M.A., Puchałka J., Fryer K.E., Martins dos Santos V.A.P. and Papin J.A. (2008) Genome-scale metabolic network analysis of the opportunistic pathogen *Pseudomonas aeruginosa* PAO1. J. Bacteriol. 190, 2790–2803 18192387 10.1128/JB.01583-07 PMC2293256

[5] Schwanemann T., Otto M., Wierckx N. and Wynands B. (2020) *Pseudomonas* as versatile aromatics cell factory. Biotechnol. J. 15, e1900569 32978889 10.1002/biot.201900569

[6] Jiménez J.I., Miñambres B., García J.L. and Díaz E. (2002) Genomic analysis of the aromatic catabolic pathways from *Pseudomonas putida* KT2440. Environ. Microbiol. 4, 824–841 12534466 10.1046/j.1462-2920.2002.00370.x

[7] Riveros-Rosas H., Julián-Sánchez A., Moreno-Hagelsieb G. and Muñoz-Clares R.A. (2019) Aldehyde dehydrogenase diversity in bacteria of the *Pseudomonas* genus. Chem. Biol. Interact. 304, 83–87 30862475 10.1016/j.cbi.2019.03.006

[8] Chowdhury P.P., Sarkar J., Basu S. and Dutta T.K. (2014) Metabolism of 2-hydroxy-1-naphthoic acid and naphthalene via gentisic acid by distinctly different sets of enzymes in *Burkholderia* sp. strain BC1. Microbiology (N. Y.) 160, 892–902 24554759 10.1099/mic.0.077495-0

[9] Nichols N.N. and Mertens J.A. (2008) Identification and transcriptional profiling of *Pseudomonas putida* genes involved in furoic acid metabolism. FEMS Microbiol. Lett. 284, 52–57 18492059 10.1111/j.1574-6968.2008.01196.x

[10] Gasteiger E., Hoogland C., Gattiker A., Duvaud S., Wilkins M.R., Appel R.D. et al. (2005) Protein identification and analysis tools on the ExPASy server. In The Proteomics Protocols Handbook(Walker J.M., ed.), pp. 571–607, Humana Press, Totowa, NJ 10.1385/1-59259-890-0:571

[11] Cardona‐Cardona Y.V., Regla I., Juárez‐Díaz J.A., Carrillo‐Campos J., López‐Ortiz M., Aguilera‐Cruz A. et al. (2022) The critical role of the aldehyde dehydrogenase PauC in spermine, spermidine, and diaminopropane toxicity in *Pseudomonas aeruginosa:* its possible use as a drug target. FEBS J. 289, 2685–2705 34767295 10.1111/febs.16277

[12] Fernández-Silva A., Juárez-Vázquez A.L., González-Segura L., Juárez-Díaz J.A. and Muñoz-Clares R.A. (2023) The uncharacterized *Pseudomonas aeruginosa* PA4189 is a novel and efficient aminoacetaldehyde dehydrogenase. Biochem. J. 480, 259–281 36727473 10.1042/BCJ20220567

[13] Dearden J.C. and Forbes W.F. (1959) Light absorption studies: part xiv. the ultraviolet absorption spectra of phenols. Can. J. Chem. 37, 1294–1304 10.1139/v59-193

[14] Muñoz-Clares R.A. and Casanova-Figueroa K. (2019) The importance of assessing aldehyde substrate inhibition for the correct determination of kinetic parameters and mechanisms: the case of the ALDH enzymes. Chem. Biol. Interact. 305, 86–97 30928398 10.1016/j.cbi.2019.03.024

[15] Kamimura N., Goto T., Takahashi K., Kasai D., Otsuka Y., Nakamura M. et al. (2017) A bacterial aromatic aldehyde dehydrogenase critical for the efficient catabolism of syringaldehyde. Sci. Rep. 7, 44422 28294121 10.1038/srep44422 PMC5353671

[16] Cleland W. (1963) The kinetics of enzyme-catalyzed reactions with two or more substrates or products I. Nomenclature and rate equations. Biochim. Biophys. Acta 67, 104–137 14021667 10.1016/0926-6569(63)90211-6

[17] Segel I.H. (1975) Enzyme Kinetics: Behavior and Analysis of Rapid Equilibrium and Steady-state Enzyme Systems, John Wiley & Sons, New York

[18] Henehan G.T.M. and Tipton K.F. (1992) Steady-state kinetic analysis of aldehyde dehydrogenase from human erythrocytes. Biochem. J. 287, 145–150 1417767 10.1042/bj2870145 PMC1133136

[19] Marchal S., Rahuel-Clermont S. and Branlant G. (2000) Role of glutamate-268 in the catalytic mechanism of nonphosphorylating glyceraldehyde-3-phosphate dehydrogenase from *Streptococcus mutans*. Biochemistry 39, 3327–3335 10727225 10.1021/bi9914208

[20] Jang E.H., Park S.A., Chi Y.M. and Lee K.S. (2015) Structural insight into the substrate inhibition mechanism of NADP^+^-dependent succinic semialdehyde dehydrogenase from *Streptococcus pyogenes*. Biochem. Biophys. Res. Commun. 461, 487–493 25888791 10.1016/j.bbrc.2015.04.047

[21] Ni L., Sheikh S. and Weiner H. (1997) Involvement of glutamate 399 and lysine 192 in the mechanism of human liver mitochondrial aldehyde dehydrogenase. J. Biol. Chem. 272, 18823–18826 9228057 10.1074/jbc.272.30.18823

[22] Perozich J., Nicholas H., Wang B., Lindahl R. and Hempel J. (1999) Relationships within the aldehyde dehydrogenase extended family. Protein Sci. 8, 137–146 10210192 10.1110/ps.8.1.137 PMC2144113

[23] Kitchen D.B., Decornez H., Furr J.R. and Bajorath J. (2004) Docking and scoring in virtual screening for drug discovery: methods and applications. Nat. Rev. Drug Discov. 3, 935–949 15520816 10.1038/nrd1549

[24] Ferreira L., Dos Santos R., Oliva G. and Andricopulo A. (2015) Molecular docking and structure-based drug design strategies. Molecules 20, 13384–13421 26205061 10.3390/molecules200713384 PMC6332083

[25] Genheden S. and Ryde U. (2015) The MM/PBSA and MM/GBSA methods to estimate ligand-binding affinities. Expert Opin. Drug Discov. 10, 449–461 25835573 10.1517/17460441.2015.1032936 PMC4487606

[26] Khan S.I., Zarin A., Ahmed S., Hasan F., Belduz A.O., Çanakçi S. et al. (2022) Degradation of lignin by *Bacillus altitudinis* SL7 isolated from pulp and paper mill effluent. Water Sci. Technol. 85, 420–432 35050893 10.2166/wst.2021.610

[27] Artigot M.P., Loiseau N., Laffitte J., Mas-Reguieg L., Tadrist S., Oswald I.P. et al. (2009) Molecular cloning and functional characterization of two CYP619 cytochrome P450s involved in biosynthesis of patulin in *Aspergillus clavatus*. Microbiology (N. Y.) 155, 1738–1747 19383676 10.1099/mic.0.024836-0 PMC2889413

[28] Jørgensen S.H., Frandsen R.J.N., Nielsen K.F., Lysøe E., Sondergaard T.E., Wimmer R. et al. (2014) *Fusarium graminearum* PKS14 is involved in orsellinic acid and orcinol synthesis. Fungal Genet. Biol. 70, 24–31 25011010 10.1016/j.fgb.2014.06.008

[29] Snini S.P., Tadrist S., Laffitte J., Jamin E.L., Oswald I.P. and Puel O. (2014) The gene PatG involved in the biosynthesis pathway of patulin, a food-borne mycotoxin, encodes a 6-methylsalicylic acid decarboxylase. Int. J. Food Microbiol. 171, 77–83 24334092 10.1016/j.ijfoodmicro.2013.11.020

[30] Brinkmann U. and Reineke W. (1992) Degradation of chlorotoluenes by *in vivo* constructed hybrid strains: problems of enzyme specificity, induction and prevention of meta-pathway. FEMS Microbiol. Lett. 75, 81–87 1526468 10.1111/j.1574-6968.1992.tb05397.x

[31] Rhys-Williams W., Taylor S.C. and Williams P.A. (1993) A novel pathway for the catabolism of 4-nitrotoluene by *Pseudomonas*. J. Gen. Microbiol. 139, 1967–1972 8245826 10.1099/00221287-139-9-1967

[32] Xia Z., Zhang W., Lei L., Liu X. and Wei H.-L. (2015) Genome-wide investigation of the genes involved in nicotine metabolism in *Pseudomonas putida* J5 by Tn5 transposon mutagenesis. Appl. Microbiol. Biotechnol. 99, 6503–6514 25808517 10.1007/s00253-015-6529-x

[33] Wang H.H., Yin B., Peng X.X., Wang J.Y., Xie Z.H., Gao J. et al. (2012) Biodegradation of nicotine by newly isolated *Pseudomonas* sp. CS3 and its metabolites. J. Appl. Microbiol. 112, 258–268 22129149 10.1111/j.1365-2672.2011.05208.x

[34] Lu S., Wang J., Chitsaz F., Derbyshire M.K., Geer R.C., Gonzales N.R. et al. (2020) CDD/SPARCLE: the conserved domain database in 2020. Nucleic. Acids. Res. 48, D265–D268 31777944 10.1093/nar/gkz991 PMC6943070

[35] Vasiliou V., Bairoch A., Tipton K.F. and Nebert D.W. (1999) Eukaryotic aldehyde dehydrogenase (ALDH) genes: Human polymorphisms, and recommended nomenclature based on divergent evolution and chromosomal mapping. Pharmacogenetics 9, 421–434 10780262

[36] Jackson B., Brocker C., Thompson D.C., Black W., Vasiliou K., Nebert D.W. et al. (2011) Update on the aldehyde dehydrogenase gene (ALDH) superfamily. Hum. Genomics 5, 283 21712190 10.1186/1479-7364-5-4-283 PMC3392178

[37] Riveros-Rosas H., González-Segura L., Julián-Sánchez A., Díaz-Sánchez Á.G. and Muñoz-Clares R.A. (2013) Structural determinants of substrate specificity in aldehyde dehydrogenases. Chem. Biol. Interact. 202, 51–61 23219887 10.1016/j.cbi.2012.11.015

[38] Ausubel F.M., Brent R., Kingston R.E., Moore D.D., Seidman J.G., Smith J.A. et al. (1992) Short Protocols in Molecular Biology: A Compendium of Methods from Current Protocols in Molecular Biology, 2nd edn, Greene Publishing Associates and John Wiley & Sons, New York

[39] Laemmli U.K. (1970) Cleavage of structural proteins during the assembly of the head of bacteriophage T4. Nature 227, 680–685 5432063 10.1038/227680a0

[40] Andrews P. (1964) Estimation of the molecular weights of proteins by Sephadex gel-filtration. Biochem. J. 91, 222–233 4158310 10.1042/bj0910222 PMC1202876

[41] Horecker B.L. and Kornberg A. (1948) The extinction coefficients of the reduced band of pyridine nucleotides. J. Biol. Chem. 175, 385–390 18873313 10.1016/S0021-9258(18)57268-9

[42] Dawson R.M.C., Elliott D.C., Elliott W.H. and Jones K.M. (1986) Data for Biochemical Research, 3rd edn, Clarendon Press, Oxford

[43] Jumper J., Evans R., Pritzel A., Green T., Figurnov M., Ronneberger O. et al. (2021) Highly accurate protein structure prediction with AlphaFold. Nature 596, 583–589 34265844 10.1038/s41586-021-03819-2 PMC8371605

[44] Halavaty A.S., Minasov G., Chen C., Joo J.C., Yakunin A.F. and Anderson W.F. (2015) 2.11 Angstrom resolution crystal structure of betaine aldehyde dehydrogenase (betB) P449M/Y450L double mutant from *Staphylococcus aureus* in complex with NAD^+^ and BME-modified Cys289. 10.2210/pdb5ez4/pdb Protein Data Bank PDB ID: 5EZ4

[45] Kim S., Chen J., Cheng T., Gindulyte A., He J., He S. et al. (2025) PubChem 2025 update. Nucleic. Acids Res. 53, D1516–D1525 39558165 10.1093/nar/gkae1059 PMC11701573

[46] Hanwell M.D., Curtis D.E., Lonie D.C., Vandermeersch T., Zurek E. and Hutchison G.R. (2012) Avogadro: an advanced semantic chemical editor, visualization, and analysis platform. J. Cheminform. 4, 17 22889332 10.1186/1758-2946-4-17 PMC3542060

[47] Serjeant E.P. and Boyd D.(eds). (1979) Ionisation Constants of Organic Acids in Aqueous Solution, IUPAC Chemical Data Series, Pergamon Press, New York

[48] Trott O. and Olson A.J. (2010) AutoDock Vina: improving the speed and accuracy of docking with a new scoring function, efficient optimization, and multithreading. J. Comput. Chem. 31, 455–461 19499576 10.1002/jcc.21334 PMC3041641

[49] Eberhardt J., Santos-Martins D., Tillack A.F. and Forli S. (2021) AutoDock Vina 1.2.0: new docking methods, expanded force field, and Python bindings. J. Chem. Inf. Model. 61, 3891–3898 34278794 10.1021/acs.jcim.1c00203 PMC10683950

[50] Pettersen E.F., Goddard T.D., Huang C.C., Couch G.S., Greenblatt D.M., Meng E.C. et al. (2004) UCSF Chimera—a visualization system for exploratory research and analysis. J. Comput. Chem. 25, 1605–1612 15264254 10.1002/jcc.20084

[51] Meng E.C., Pettersen E.F., Couch G.S., Huang C.C. and Ferrin T.E. (2006) Tools for integrated sequence-structure analysis with UCSF Chimera. BMC Bioinformatics 7, 339 16836757 10.1186/1471-2105-7-339 PMC1570152

[52] Jo S., Kim T., Iyer V.G. and Im W. (2008) CHARMM-GUI: a web-based graphical user interface for CHARMM. J. Comput. Chem. 29, 1859–1865 18351591 10.1002/jcc.20945

[53] Lee J., Cheng X., Swails J.M., Yeom M.S., Eastman P.K., Lemkul J.A. et al. (2016) CHARMM-GUI input generator for NAMD, GROMACS, AMBER, OpenMM, and CHARMM/OpenMM simulations using the CHARMM36 additive force field. J. Chem. Theory Comput. 12, 405–413 26631602 10.1021/acs.jctc.5b00935 PMC4712441

[54] Huang J., Rauscher S., Nawrocki G., Ran T., Feig M., de Groot B.L. et al. (2017) CHARMM36m: an improved force field for folded and intrinsically disordered proteins. Nat. Methods 14, 71–73 27819658 10.1038/nmeth.4067 PMC5199616

[55] James M., Murtola T., Schulz R., Smith J.C., Hess B. and Lindahl E. (2015) GROMACS: high performance molecular simulations through multi-level parallelism from laptops to supercomputers. Software X 2, 19–25 10.1016/j.softx.2015.06.001

[56] Bürgi H.B., Dunitz J.D. and Shefter E. (1973) Geometrical reaction coordinates. II. Nucleophilic addition to a carbonyl group. J. Am. Chem. Soc. 95, 5065–5067 10.1021/ja00796a058

[57] Fernández I., Bickelhaupt F.M. and Svatunek D. (2023) Unraveling the Bürgi–Dunitz angle with precision: the power of a two-dimensional energy decomposition analysis. J. Chem. Theory Comput. 19, 7300–7306 37791978 10.1021/acs.jctc.3c00907 PMC10601473

[58] Bruice T.C. and Lightstone F.C. (1999) Ground state and transition state contributions to the rates of intramolecular and enzymatic reactions. Acc. Chem. Res. 32, 127–136 10.1021/ar960131y

[59] Hur S. and Bruice T.C. (2003) The near attack conformation approach to the study of the chorismate to prephenate reaction. Proc. Natl. Acad. Sci. U.S.A. 100, 12015–12020 14523243 10.1073/pnas.1534873100 PMC218705

[60] Chodera J.D. and Noé F. (2014) Markov state models of biomolecular conformational dynamics. Curr. Opin. Struct. Biol. 25, 135–144 24836551 10.1016/j.sbi.2014.04.002 PMC4124001

[61] Prada-Gracia D., Gómez-Gardeñes J., Echenique P. and Falo F. (2009) Exploring the free energy landscape: from dynamics to networks and back. PLoS Comput. Biol. 5, e1000415 19557191 10.1371/journal.pcbi.1000415 PMC2694367

[62] Kollman P.A., Massova I., Reyes C., Kuhn B., Huo S., Chong L. et al. (2000) Calculating structures and free energies of complex molecules: combining molecular mechanics and continuum models. Acc. Chem. Res. 33, 889–897 11123888 10.1021/ar000033j

[63] Valdés-Tresanco M.S., Valdés-Tresanco M.E., Valiente P.A. and Moreno E. (2021) gmx_MMPBSA: a new tool to perform end-state free energy calculations with GROMACS. J. Chem. Theory Comput. 17, 6281–6291 34586825 10.1021/acs.jctc.1c00645

[64] Miller B.R., McGee T.D., Swails J.M., Homeyer N., Gohlke H. and Roitberg A.E. (2012) *MMPBSA.py*: an efficient program for end-state free energy calculations. J. Chem. Theory Comput. 8, 3314–3321 26605738 10.1021/ct300418h

[65] Moreno-Hagelsieb G., Wang Z., Walsh S. and ElSherbiny A. (2013) Phylogenomic clustering for selecting non-redundant genomes for comparative genomics. Bioinformatics 29, 947–949 23396122 10.1093/bioinformatics/btt064

[66] Altschul S., Madden T.L., Schäffer A.A., Zhang J., Zhang Z., Miller W. et al. (1997) Gapped BLAST and PSI-BLAST: a new generation of protein database search programs. Nucleic. Acids Res. 25, 3389–3402 9254694 10.1093/nar/25.17.3389 PMC146917

[67] O'Leary N.A., Wright M.W., Brister J.R., Ciufo S., Haddad D., McVeigh R. et al. (2016) Reference sequence (RefSeq) database at NCBI: current status, taxonomic expansion, and functional annotation. Nucleic. Acids Res. 44, D733–D745 26553804 10.1093/nar/gkv1189 PMC4702849

[68] Katoh K. and Standley D.M. (2013) MAFFT multiple sequence alignment software version 7: improvements in performance and usability. Mol. Biol. Evol. 30, 772–780 23329690 10.1093/molbev/mst010 PMC3603318

[69] Kumar S., Stecher G., Suleski M., Sanderford M., Sharma S. and Tamura K. (2024) MEGA12: molecular evolutionary genetic analysis version 12 for adaptive and green computing. Mol. Biol. Evol. 41, msae263 39708372 10.1093/molbev/msae263 PMC11683415

[70] Le S.Q. and Gascuel O. (2008) An improved general amino acid replacement matrix. Mol. Biol. Evol. 25, 1307–1320 18367465 10.1093/molbev/msn067

[71] Felsenstein J. (1985) Confidence limits on phylogenies: an approach using the bootstrap. Evolution (N. Y.) 39, 783–791 28561359 10.1111/j.1558-5646.1985.tb00420.x

[72] Saitou N. and Nei M. (1987) The neighbor-joining method: a new method for reconstructing phylogenetic trees. Mol. Biol. Evol. 4, 406–425 3447015 10.1093/oxfordjournals.molbev.a040454

[73] Crooks G.E., Hon G., Chandonia J.-M. and Brenner S.E. (2004) WebLogo: a sequence logo generator. Genome Res. 14, 1188–1190 15173120 10.1101/gr.849004 PMC419797

