# Supplementary material for: Kinetic, computational, and phylogenetic characterisation of PA2125 from Pseudomonas aeruginosa PAO1: a hydroxybenzaldehyde dehydrogenase of the newly defined ALDH33 family
Source: Biosci Rep. 2026 Sep 22;46(10):BSR20260546. doi: 10.1042/BSR20260546 (PMC13598318; doi:10.1042/BSR20260546)
Supplement: Supplementary file 1 [file BSR-2026-0546-T_supp.pdf]

Supplementary Material

Kinetic, computational, and phylogenetic characterisation of PA2125 from *Pseudomonas aeruginosa* PAO1: A hydroxybenzaldehyde dehydrogenase of the newly defined ALDH33 family

Gustavo A. Valero-Pibaral[1,†], Alexey Llopiz[1,†], Carlos A. Rueda-Sanabria[1,a], Héctor Riveros-Rosas[2], Arline Fernández-Silva[1], Carlos Mújica-Jiménez[1], and Rosario A. Muñoz-Clares[1,*]

[1]Departamento de Bioquímica, Facultad de Química. [2]Departamento de Bioquímica, Facultad de Medicina. Universidad Nacional Autónoma de México, Ciudad de México, 04510, México.

[†]These authors contributed equally to this work.

[a]Present address: Facultad de Ingenierías, Universidad Surcolombiana, Neiva, Huila, Colombia.

[*]Corresponding author: Rosario A. Muñoz-Clares, Ciudad Universitaria, C.P. 04510, Ciudad de México, México. e-mail: clares@unam.mx

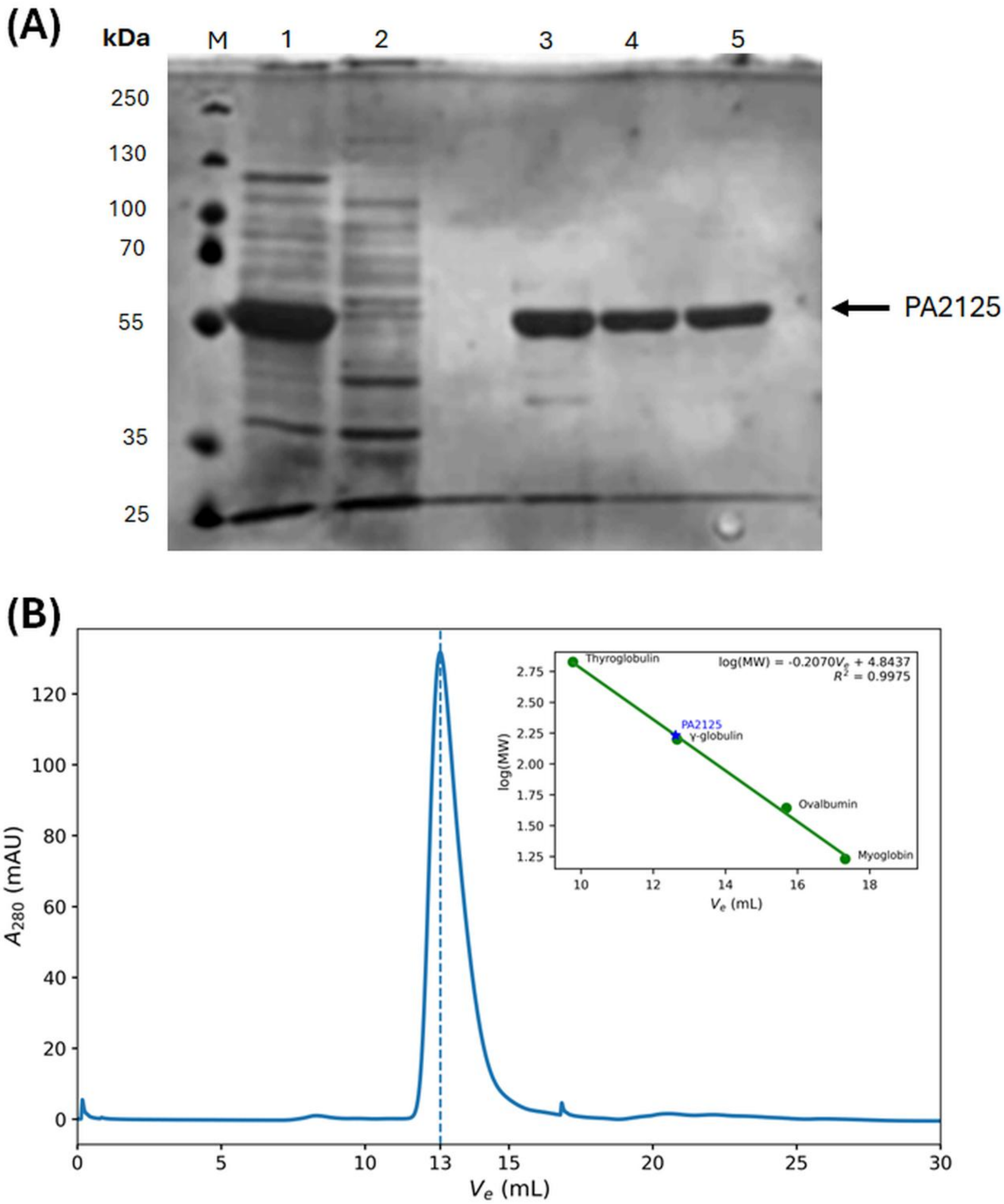

Figure S1. Purification and estimation of the apparent native molecular mass and oligomeric state of PA2125.

(A) SDS-PAGE showing the results of PA2125 purification. Lanes: M, molecular mass markers; 1, supernatant of crude cell extracts; 2, flow-through fraction; 3 to 5, eluted fractions. **(B)** Elution profile of PA2125 from size-exclusion chromatography. The dashed vertical line indicates the peak elution volume (V_e) corresponding to an apparent molecular mass of approximately 171 kDa. Inset: calibration curve generated using the indicated molecular-mass standards.

Table S1. Molar absorptivities at 340 nm of selected aromatic aldehydes.

Aldehyde (abbreviation)	ε_{340}[a] (M^{-1} cm^{-1})
2-hydroxybenzaldehyde (2-OHBZAL; salicylaldehyde)	2,530
3-hydroxybenzaldehyde (3-OHBZAL)	1,180
4-hydroxybenzaldehyde (4-OHBZAL)	8,396
2,3-dihydroxybenzaldehyde (2,3-diOHBZAL)	1,994
2,3-dihydroxybenzoate (2,3-diOHBZA)	270
2,4-dihydroxybenzaldehyde (2,4-diOHBZAL)	6,609
2,5-dihydroxybenzaldehyde (2,5-diOHBZAL; gentisaldehyde; GSA)	2,215
2,5-dihydroxybenzoate (2,5-diOHBZA; gentisate)	1,680
2,6-dihydroxybenzaldehyde (2,6-diOHBZAL)	1,133
2,6-dihydroxybenzoate (2,6-diOHBZA)	126
3,4-dihydroxybenzaldehyde (3,4-diOHBZAL)	21,155
3,5-dihydroxybenzaldehyde (3,5-diOHBZAL)	1,862
2,3,4-trihydroxybenzaldehyde (2,3,4-triOHBZAL)	17,357
2,4,5-trihydroxybenzaldehyde (2,4,5-triOHBZAL)	15,524
2,4,6-trihydroxybenzaldehyde (2,4,6-triOHBZAL)	8,166
3,4,5-trihydroxybenzaldehyde (3,4,5-triOHBZAL)	11,649
4-hydroxy-3-methoxybenzaldehyde (4-OH-3-MeOBZAL; vanillin)	25,964
2-methoxybenzaldehyde (2-MeOBZAL)	2,750
3-methoxybenzaldehyde (3-MeOBZAL)	620
1-naphthaldehyde (1-NaphAL)	4,780
2-naphthaldehyde (2-NaphAL)	1,600

Only compounds with ε_{340} values greater than 1 % of the molar absorptivity of NADH at 340 nm (ε_{340} = 6,220 M^{-1} cm^{-1}) are reported. Molar absorptivities were determined at pH 8.0 in the assay buffer used for activity measurements. These ε_{340} values were used to compute the corrected molar absorptivity ($\varepsilon_{340,corr}$) according to eqn. (1).

Table S2. Specific activities of PA2125 with aliphatic, aromatic and heteroaromatic aldehydes.

Aldehyde (abbreviation)	Chemical structure	Relative specific activity (% BZAL)
**Aliphatic aldehydes**		
acetaldehyde<br>(AcAL)	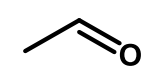	<1
chloroacetaldehyde<br>(ClAcAL)	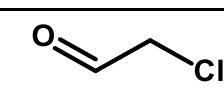	<1
propionaldehyde<br>(ProAL)	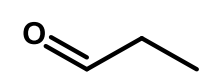	<1
butyraldehyde<br>(ButAL)	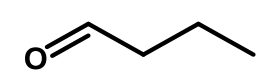	<1
pentanal<br>(PentAL)	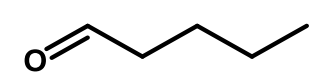	<1
hexanal<br>(HexAL)	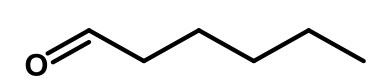	<1
cyclohexanecarboxaldehyde	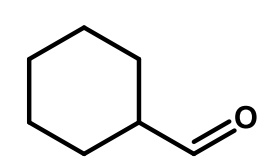	<1
2-butenal	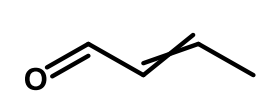	<1
(2E,4E)-2,4-hexadienal	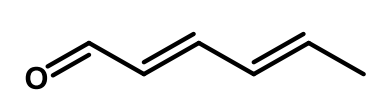	<1
3,7-dimethylocta-2,6-dienal<br>(geranial)	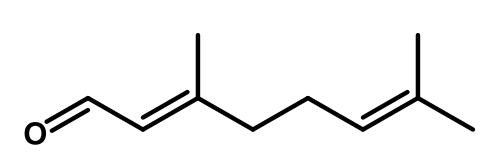	<1
**Hydroxybenzaldehydes***		
benzaldehyde<br>(BZAL)	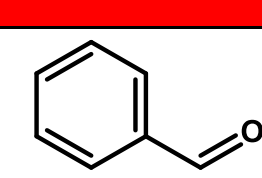	100

Table S2 (continued)

Aldehyde (abbreviation)	Chemical structure	Relative specific activity (% BZAL)
2-hydroxybenzaldehyde (2-OHBZAL, salicylaldehyde)	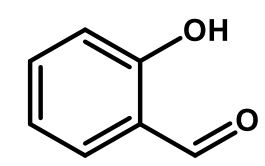	538
3-hydroxybenzaldehyde (3-OHBZAL)	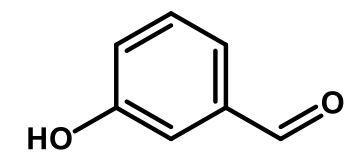	310
4-hydroxybenzaldehyde (4-OHBZAL)	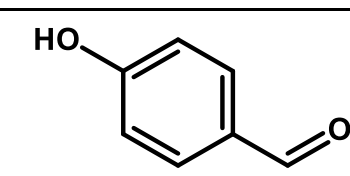	<1
2,3-dihydroxybenzaldehyde (2,3-diOHBZAL)	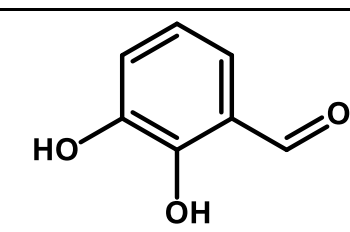	47
2,4-dihydroxybenzaldehyde (2,4-diOHBZAL)	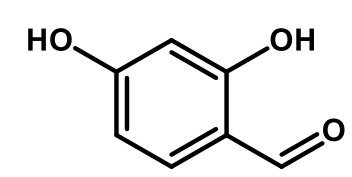	45
2,5-dihydroxybenzaldehyde (gentisaldehyde; GSA)	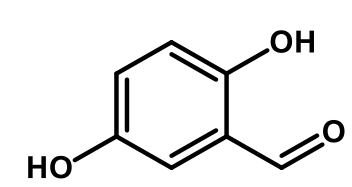	12,838
2,6-dihydroxybenzaldehyde (2,6-diOHBZAL)	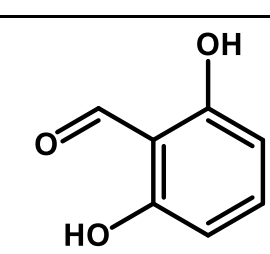	165
3,4-dihydroxybenzaldehyde (3,4-diOHBZAL)	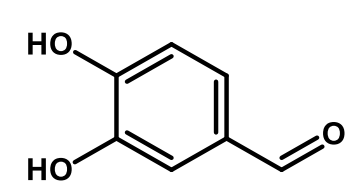	300
3,5-dihydroxybenzaldehyde (3,5-diOHBZAL)	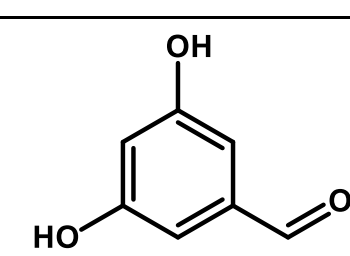	209
2,3,4-trihydroxybenzaldehyde (2,3,4-triOHBZAL)	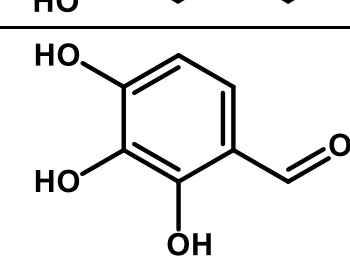	<1
2,4,5-trihydroxybenzaldehyde (2,4,5-triOHBZAL)	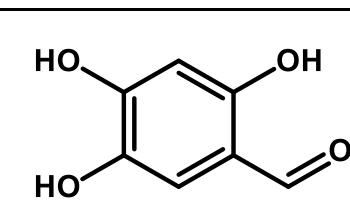	<1
2,4,6-trihydroxybenzaldehyde (2,4,6-triOHBZAL)	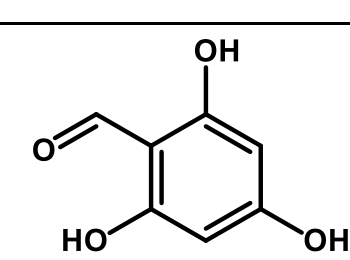	<1

Table S2 (continued)

Aldehyde (abbreviation)	Chemical structure	Relative specific activity (% BZAL)
3,4,5-trihydroxybenzaldehyde (3,4,5-triOHBZAL)	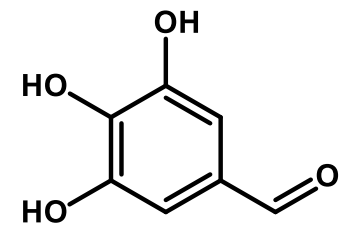	<1
4-hydroxy-3-methoxybenzaldehyde (4-OH-3-MeOBZAL, vanillin)	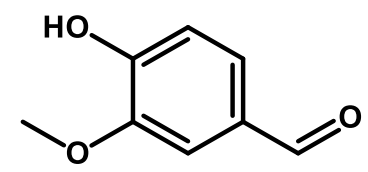	47
**Non-hydroxybenzaldehydes derivatives**		
2-methoxybenzaldehyde (2-MeOBZAL)	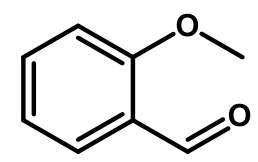	<1
3-methoxybenzaldehyde (3-MeOBZAL)	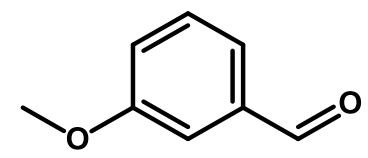	136
4-methoxybenzaldehyde (4-MeOBZAL)	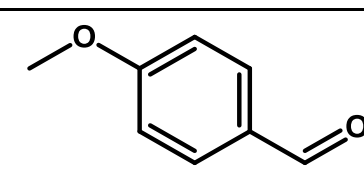	29
2-methylbenzaldehyde (2-MeBZAL)	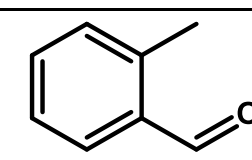	8
3-methylbenzaldehyde (3-MeBZAL)	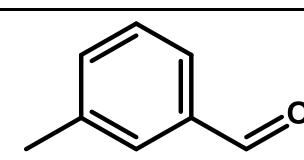	65
4-methylbenzaldehyde (4-MeBZAL)	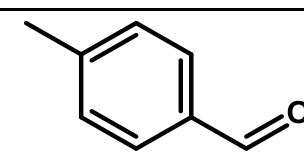	107
3-isopropylbenzaldehyde (3-iPrBZAL)	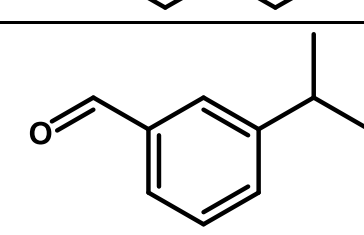	32
4-isopropylbenzaldehyde (4-iPrBZAL)	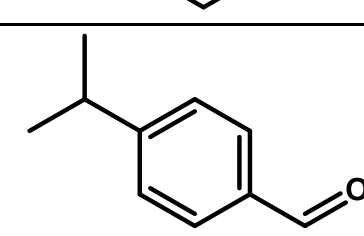	79
4-tert-butylbenzaldehyde (4-tBuBZAL)	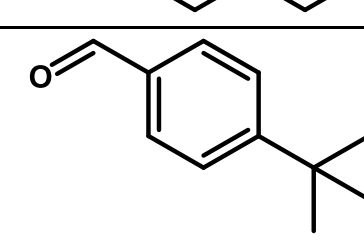	<1
2-chlorobenzaldehyde (2-ClBZAL)	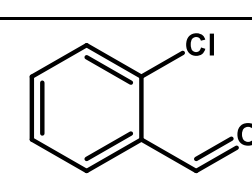	<1
3-chlorobenzaldehyde (3-ClBZAL)	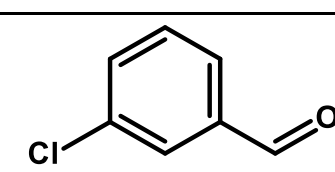	340
4-chlorobenzaldehyde (4-ClBZAL)	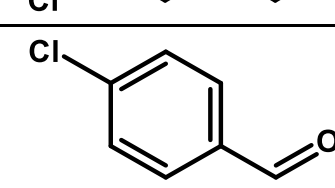	336
2-nitrobenzaldehyde (2-NO_2BZAL)	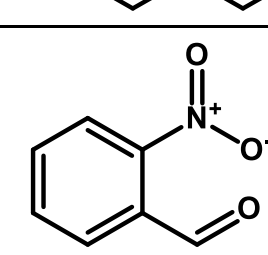	<1

Table S2 (continued)

Aldehyde (abbreviation)	Chemical structure	Relative specific activity (% BZAL)
3-nitrobenzaldehyde ($3\text{-}NO_2BZAL$)	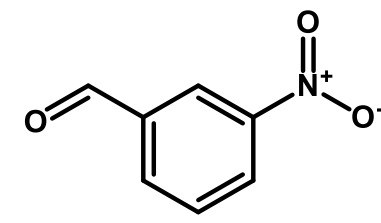	145
4-nitrobenzaldehyde ($4\text{-}NO_2BZAL$)	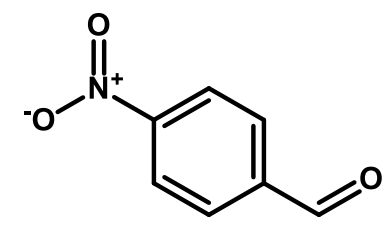	106
2-carboxybenzaldehyde (2-COOHBZAL)	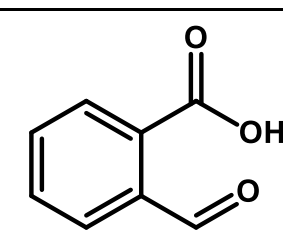	<1
3-carboxybenzaldehyde (3-COOHBZAL)	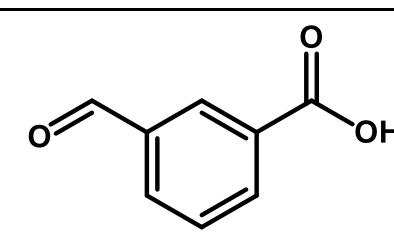	8
4-carboxybenzaldehyde (4-COOHBZAL)	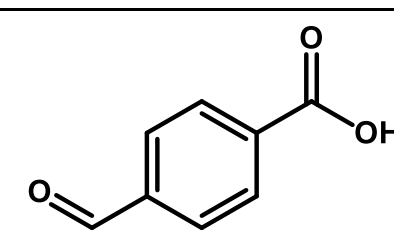	<1
**Other aromatic heteroaromatic aldehydes**		
phenylacetaldehyde (PhAcAL)	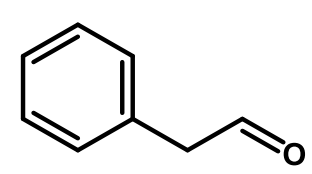	<1
3-phenylpropionaldehyde (PhPropAL)	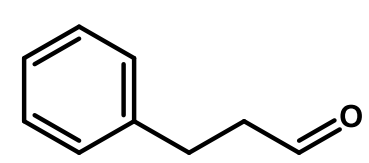	<1
^{1}H-pyrrole-2-carboxaldehyde (Pyrr2CHO)	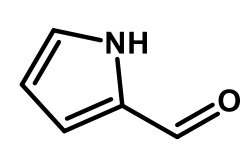	<1
^{1}H-imidazole-4-carboxaldehyde (Im4CHO)	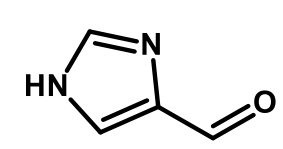	<1
1-naphthaldehyde (1-NaphAL)*	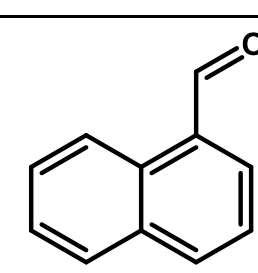	<1
2-naphthaldehyde (2-NaphAL)*	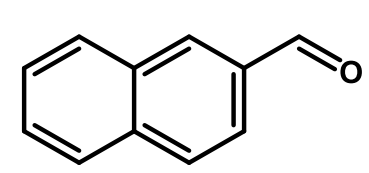	<1
furfural	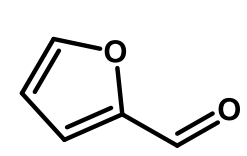	2

Table S2 (continued)

Aldehyde (abbreviation)	Chemical structure	Relative specific activity (% BZAL)
5-hydroxymethylfurfural (5-HMF)	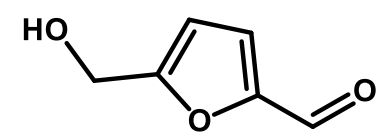	26
pyridine-2-carboxaldehyde (Pyd2CHO)	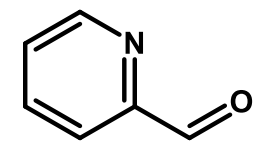	7
pyridine-3-carboxaldehyde (Pyd3CHO)	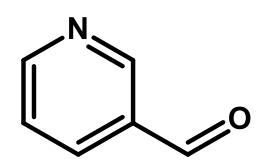	107
pyridine-4-carboxaldehyde (Pyd4CHO)	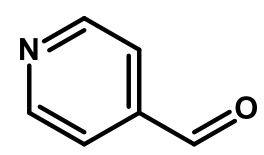	148

Enzyme activity assays were performed at 30 °C in 100 mM KH_2PO_4/K_2HPO_4, pH 8.0, with 0.6 mM NAD^+ and 1.0 mM aldehyde, by monitoring the change in absorbance at 340 nm. When the aldehyde and/or the corresponding carboxylic acid contributed more than 1% of the molar absorptivity of NADH at 340 nm, the appropriate corrected molar absorptivity was used to calculate enzyme activity. For aldehydes marked with an asterisk, assays were performed with 0.25 mM aldehyde because their high absorbance at 340 nm precluded measurements at higher concentrations. Relative activities were calculated using the specific activity with BZAL at the corresponding concentration as 100%: 5.1 and 1.5 U mg^{-1} prot. at 1.0 mM BZAL and at 0.25 mM BZAL, respectively.

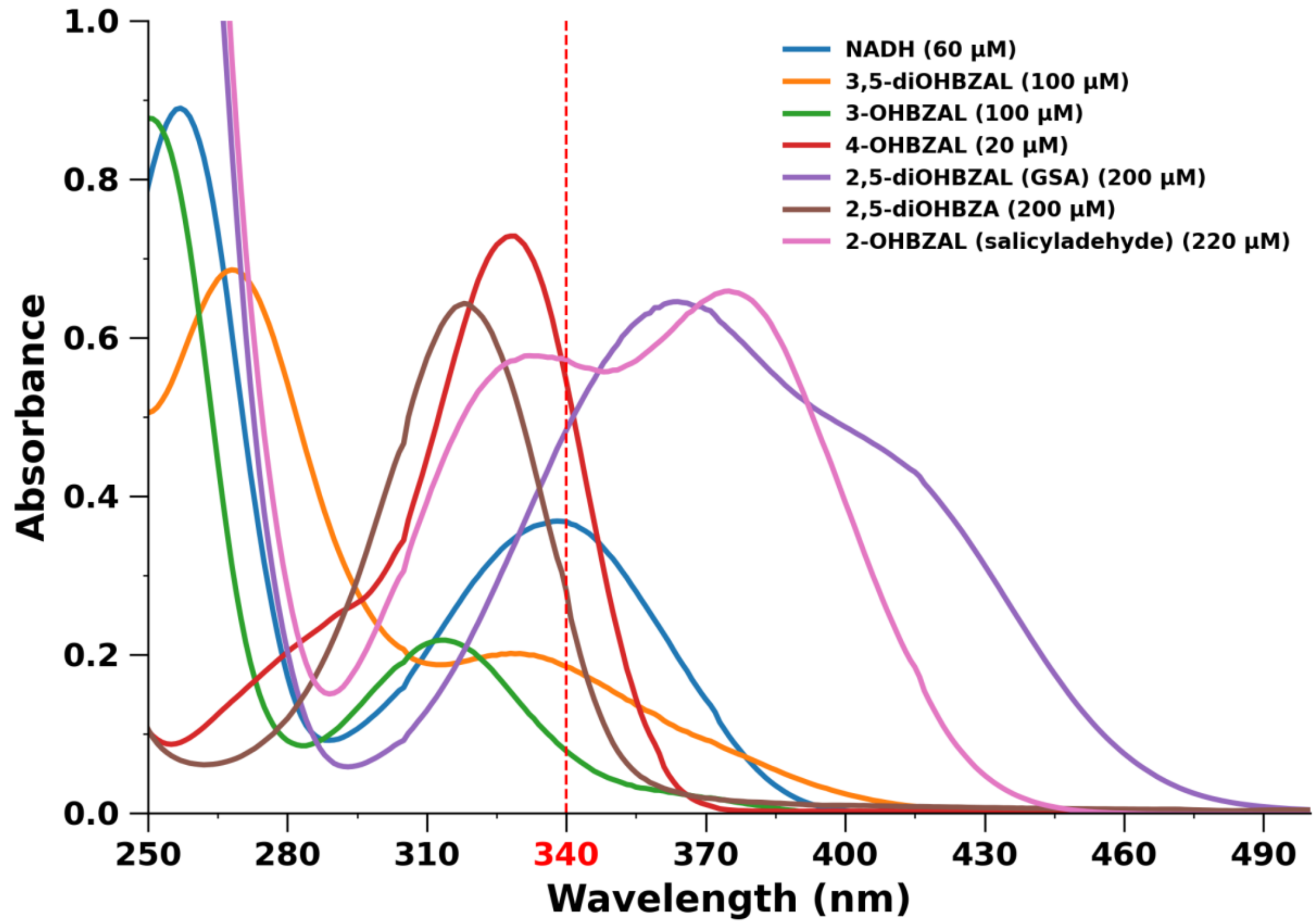

Figure S2. UV–visible absorption spectra of selected hydroxybenzaldehydes and gentisate (2,5-diOHBZA).

The spectrum of NADH was included for comparison. The dashed red line indicates 340 nm, the wavelength used to monitor NADH formation in the enzyme assays. Compound concentrations are indicated in parentheses. Spectra were recorded under the same buffer conditions employed for the kinetic measurements. Because different concentrations were used to keep absorbance values within the instrumental range, absorbance intensities should not be compared among compounds. Rather, the spectra illustrate why compound-specific ε_{340} values were required to correct the rates measured from NADH formation.

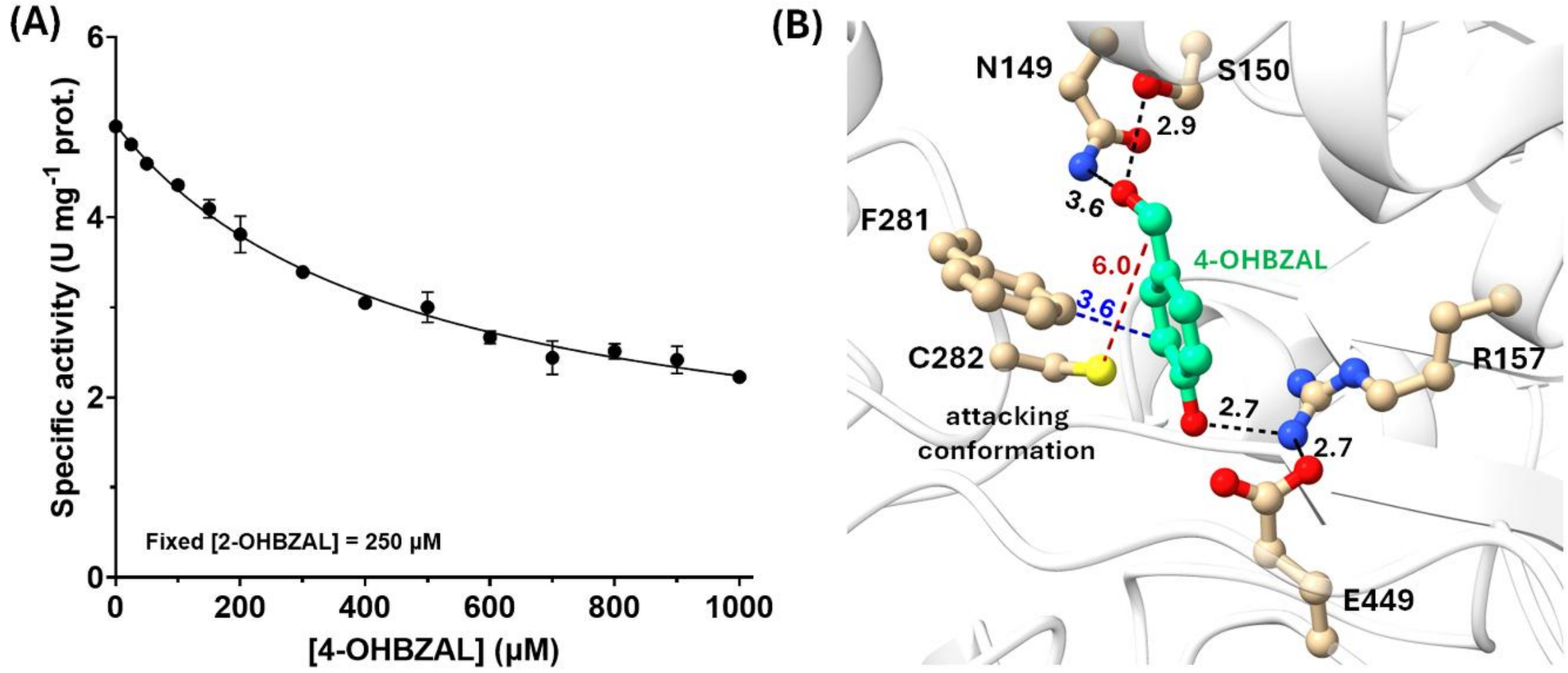

Figure S3. Inhibitory effect of 4-OHBZAL on PA2125 and predicted binding mode in the active site.

(A) Specific activity of the enzyme as a function of 4-OHBZAL concentration at a fixed 2-OHBZAL (salicylaldehyde) concentration of 250 μM. Enzyme activity assays were performed at 30 °C in 100 mM KH_2PO_4/K_2HPO_4, pH 8.0, and 0.6 mM NAD^+ by monitoring the decrease in absorbance at 400 nm, where 2-OHBZAL absorbs strongly and NADH absorbance is negligible. Data are means ± SD of at least two independent experiments. Data were fit to eqn. (4), described in the main text. R^2 value of the fit was 0.9953. **(B)** Proposed binding mode of 4-OHBZAL in the active site obtained by molecular docking. Key interacting residues and relevant interatomic distances (Å) are indicated. The inhibitor is shown in green, and active-site residues are shown in tan. The catalytic Cys282 residue is shown positioned for nucleophilic attack.

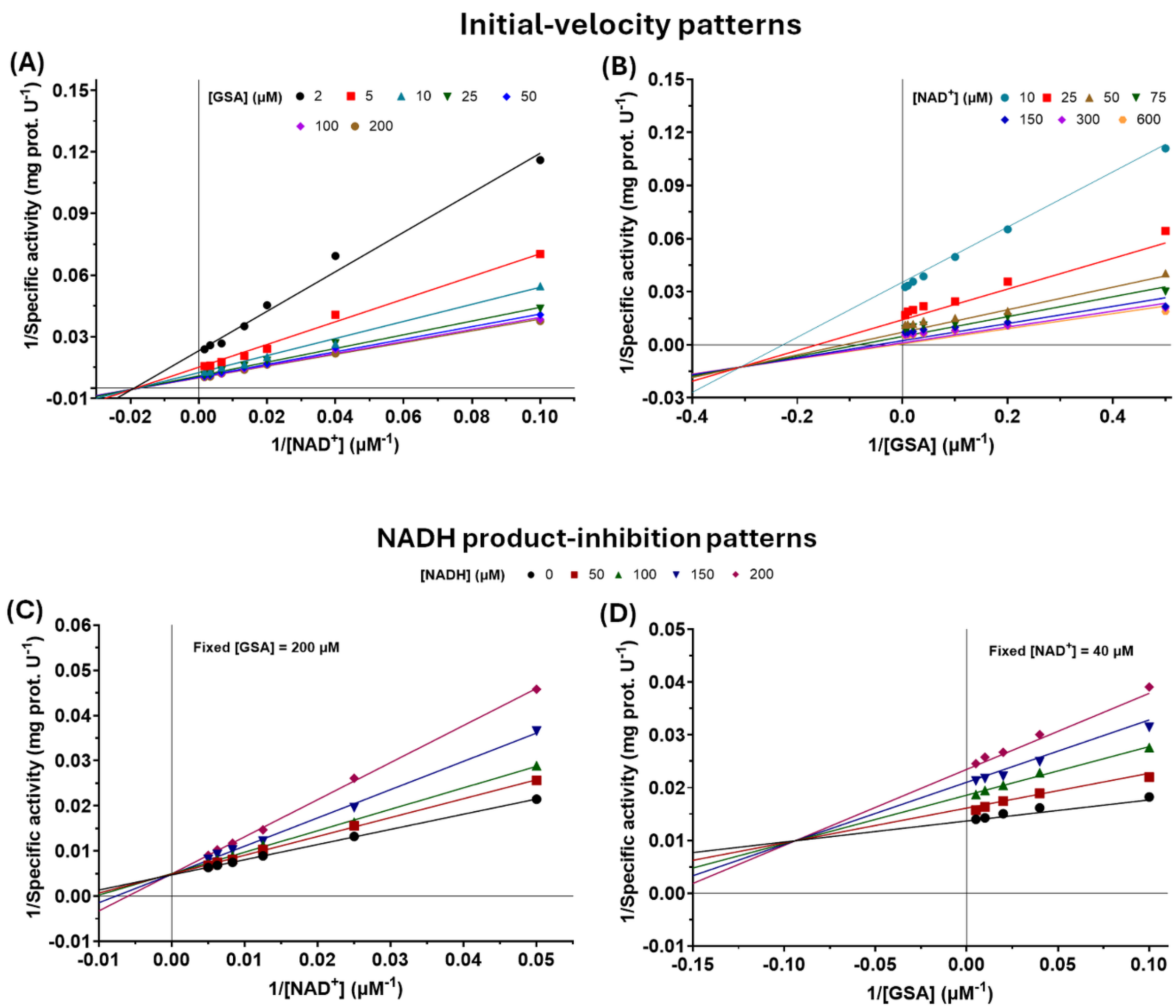

Figure S4. Lineweaver–Burk plots of initial-velocity and NADH product-inhibition patterns for the enzymatic oxidation of GSA.

(A) Initial-velocity patterns obtained by varying NAD^+ concentrations at different fixed GSA concentrations. **(B)** Initial-velocity patterns obtained by varying GSA concentrations at different fixed NAD^+ concentrations. **(C)** Product-inhibition patterns with NADH at fixed saturating GSA concentration and variable NAD^+. **(D)** Product-inhibition patterns with NADH at fixed subsaturating NAD^+ concentration and variable GSA. Lines were calculated from the parameter estimates obtained by global nonlinear fitting of the untransformed data shown in Fig. 3 (Table 2) and are shown here in double-reciprocal form only to illustrate the intersecting pattern diagnostic of an ordered sequential steady-state Bi Bi mechanism.

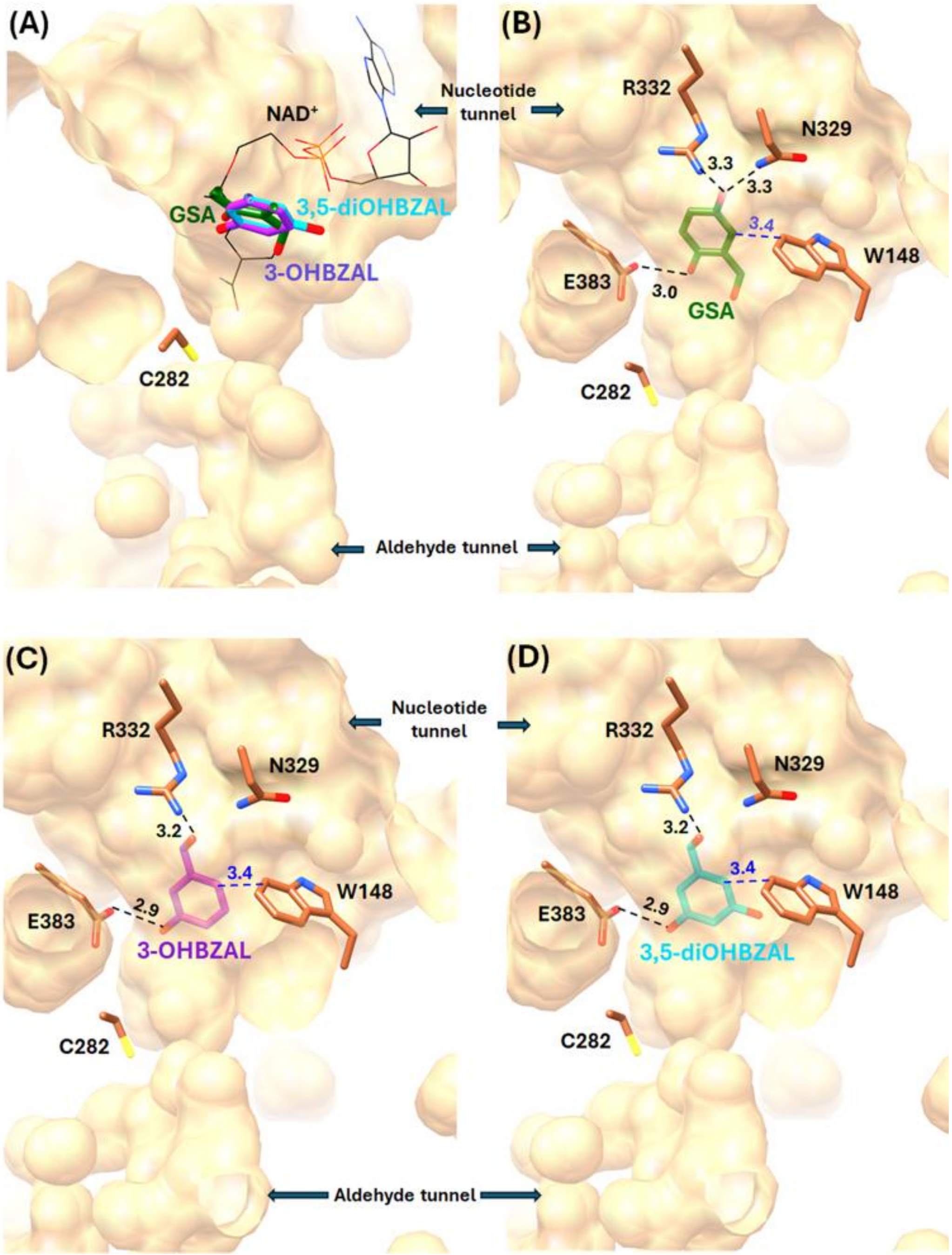

Figure S5. Representative docking poses of GSA, 3-OHBZAL, and 3,5-diOHBZAL in the nucleotide-binding site of apo PA2125.

(A) Superposition of the docked poses of GSA, 3-OHBZAL, and 3,5-diOHBZAL in the nucleotide-binding site showing the inner surface of the aldehyde and nucleotide tunnels, through which the aldehyde and nucleotide, respectively, enter the active site, the catalytic Cys282, and the reference position of NAD^+ in the PA2125•NAD^+ complex. **(B–D)** Representative poses of GSA, 3-OHBZAL, and 3,5-diOHBZAL, respectively, showing polar contacts (black dashed lines) with Arg332, Asn329, Glu383, and aromatic contacts (blue dashed lines) with Trp148. Distances are given in Å.

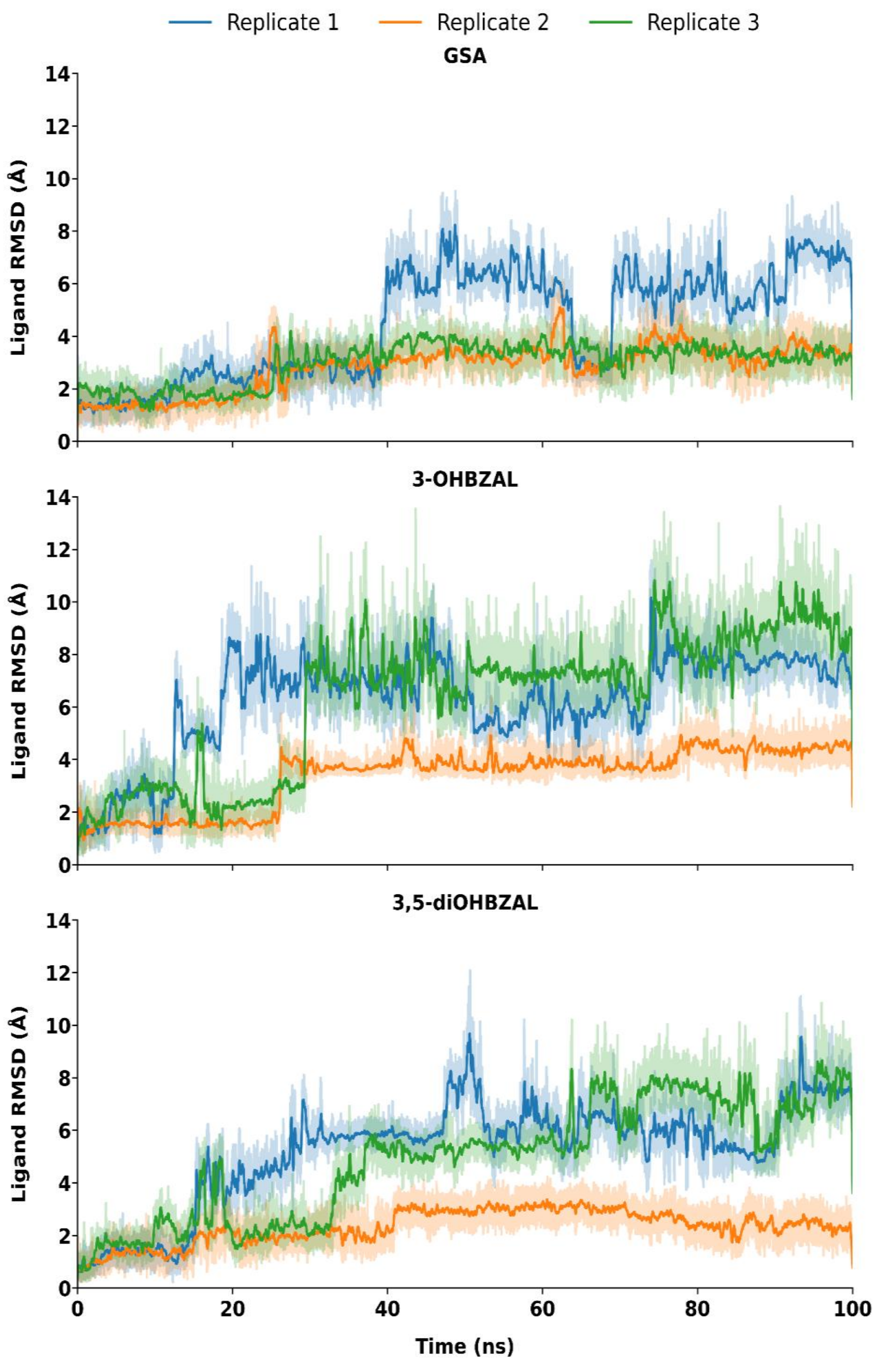

Figure S6. Molecular dynamics analysis of ternary PA2125·NAD$^+$·aldehyde complexes with GSA, 3-OHBZAL, and 3,5-diOHBZAL.

Ligand heavy-atom RMSD values were calculated along three independent 100 ns MD replicates after alignment of the protein backbone. Thin traces correspond to instantaneous RMSD values, and solid lines represent running averages calculated with a 0.2 ns window. The same color code is used for the three replicates in all panels.

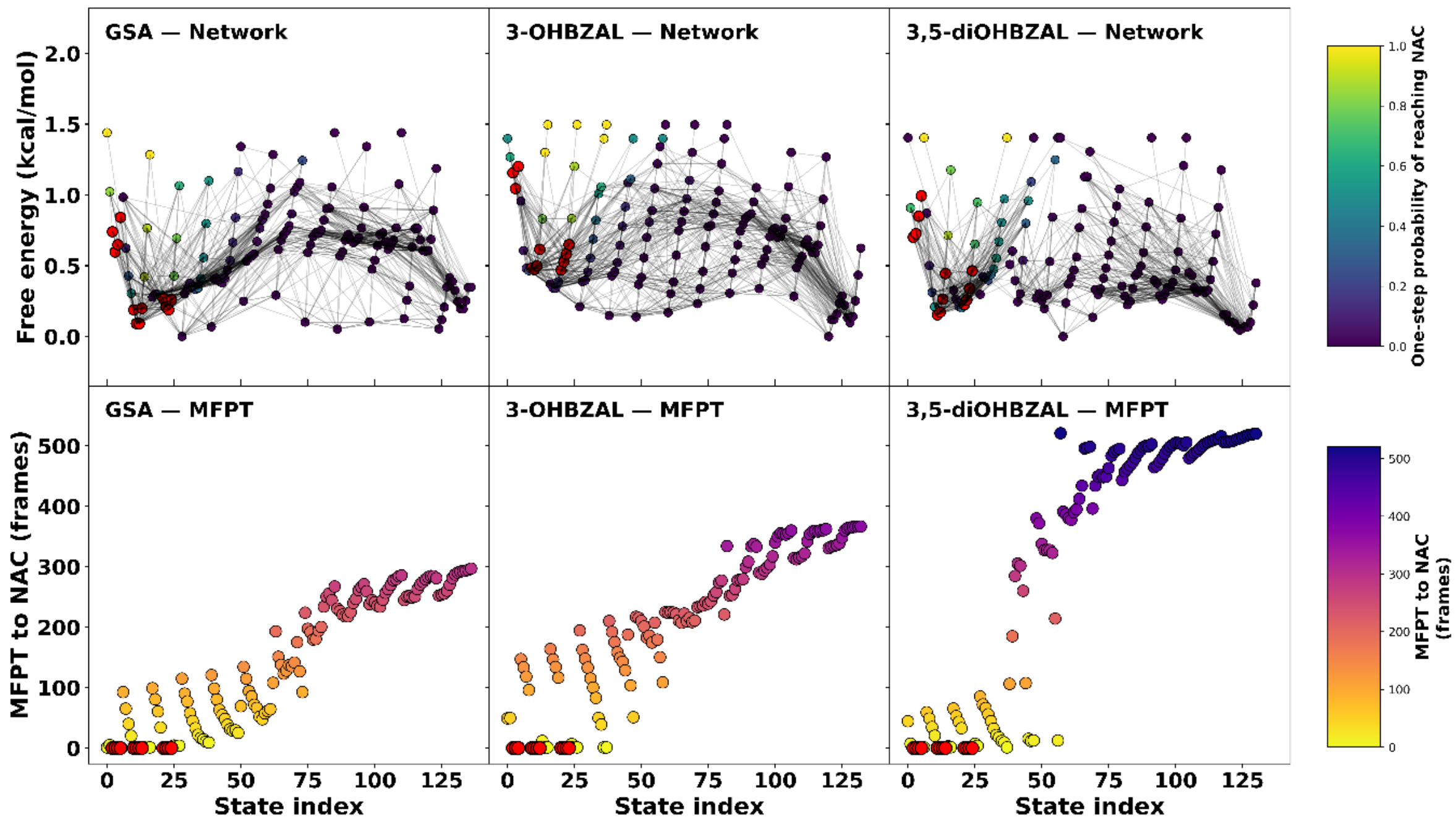

Figure S7. **Network analysis of NAC accessibility from catalytic-geometry state transitions.**

The conformational space sampled in MD simulations of ternary PA2125·NAD⁺·aldehyde complexes with GSA, 3-OHBZAL, and 3,5-diOHBZAL was analysed using the catalytic-geometry coordinates d_a and α, where d_a is the distance between the catalytic Cys282 Sγ atom and the aldehyde carbonyl carbon, and α is the Sγ–C=O angle. The d_a,α space was divided into a two-dimensional grid, and each populated grid cell was treated as a catalytic-geometry state. **Top panels** show transition networks among these states; nodes represent populated d_a,α states, edges indicate observed transitions between states, and the vertical position of each node corresponds to the relative free energy of that state calculated as described in the Materials and methods section. Node color indicates the probability that a single transition from a given state leads directly to a near attacking conformation (NAC). Only frames within d_a = 2.5–8.0 Å and α = 60–160° were included in this analysis, because states outside this region showed negligible probability of reaching NAC. Red-outlined nodes indicate NAC states. **Bottom panels** show the mean first-passage time (MFPT) from each state to NAC, expressed in frames (1 frame = ps). State indices are arbitrary labels assigned to the populated grid cells and do not represent RMSD-based structural clusters.

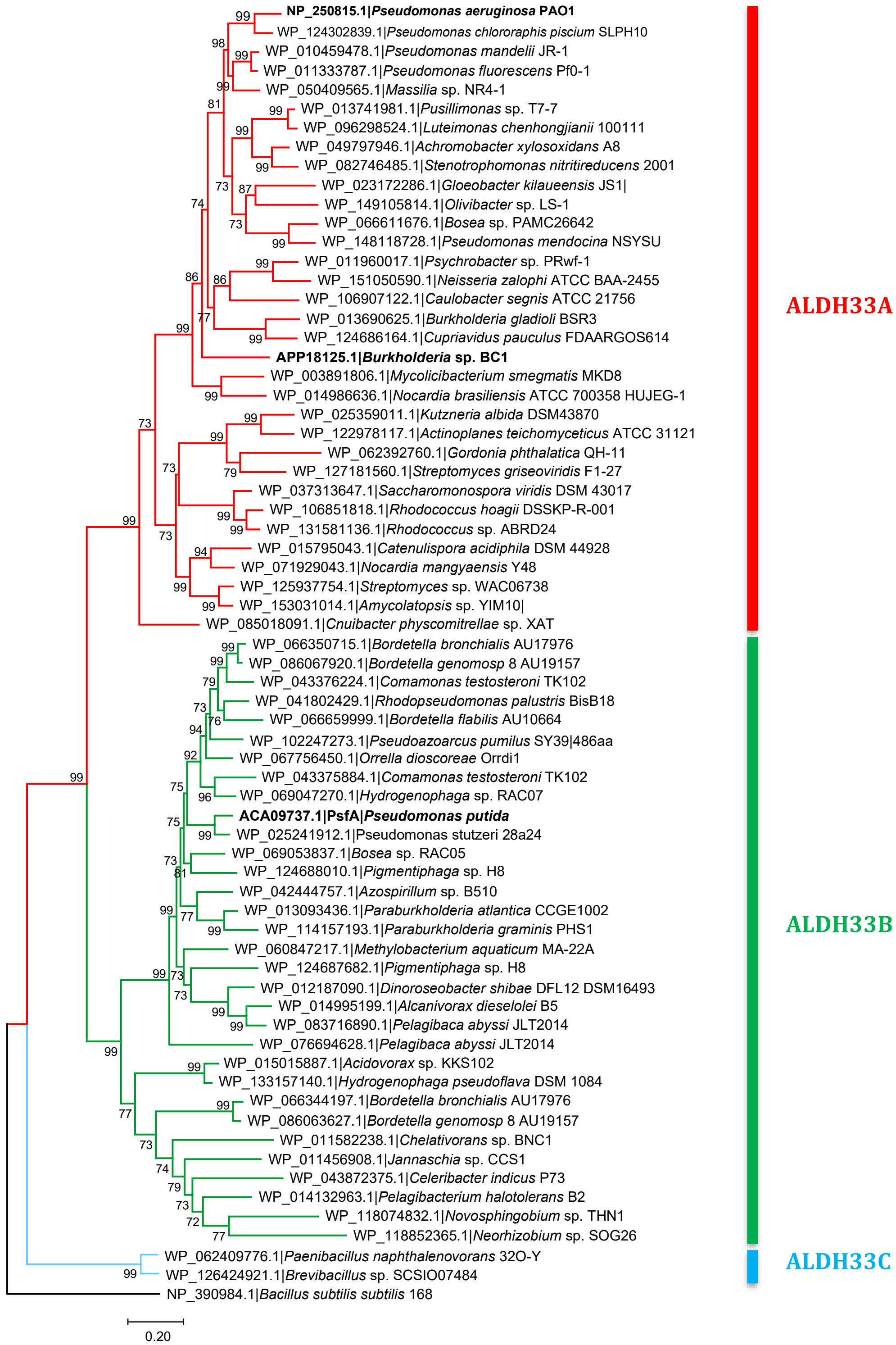

Figure S8. **Maximum-likelihood tree of ALDH33 family proteins.**

The tree was inferred with MEGA12 from 67 amino acid sequences using the LG+F+G+I model. The tree with the highest log-likelihood (−41357.45) is shown. Branch colours indicate the three ALDH33 subfamilies defined in this study. Branch lengths represent the number of amino acid substitutions per site. PA2125 from *Pseudomonas aeruginosa* PAO1 and two previously reported ALDH33-related enzymes are shown in bold. Bootstrap support values from 500 replicates are indicated at the nodes. Only bootstrap values ≥ 70% are shown. The tree was rooted using the ALDH25 GbsA sequence from *Bacillus subtilis* 168 as the outgroup. Complete sequence and taxonomic information are provided in Table S3.

Table S3: Protein sequences used for the phylogenetic analysis of PA2125-related ALDH33 enzymes. Sequences are listed according to their assignment to ALDH33A, ALDH33B or ALDH33C subfamilies. Within each category, entries are arranged alphabetically by class and within each class, by organism name. PA2125 from *Pseudomonas aeruginosa* PAO1 and the two previously reported ALDH33-related enzymes—a gentisaldehyde dehydrogenase in ALDH33A [6] and a proposed furfural dehydrogenase in ALDH33B [7]—are shown in bold. A dash indicates that no RefSeq assembly accession could be assigned to the corresponding protein sequence.

Protein accession	Protein length (aa)	RefSeq assembly accession	Organism/strain	Class
**ALDH33A**				
WP_122978117.1	508	GCF_003711105.1	*Actinoplanes teichomyceticus* ATCC 31121	Actinomycetes
WP_153031014.1	479	GCF_009429145.1	*Amycolatopsis* sp. YIM10	Actinomycetes
WP_015795043.1	479	GCF_000024025.1	*Catenulispora acidiphila* DSM 44928	Actinomycetes
WP_085018091.1	477	GCF_002096055.1	*Cnuibacter physcomitrellae* XAT	Actinomycetes
WP_062392760.1	491	GCF_001305675.1	*Gordonia phthalatica* QH-11	Actinomycetes
WP_025359011.1	490	GCF_000525635.1	*Kutzneria albida* DSM 43870	Actinomycetes
WP_003891806.1	480	GCF_000331165.2	*Mycolicibacterium smegmatis* MKD8	Actinomycetes
WP_014986636.1	488	GCF_000250675.2	*Nocardia brasiliensis* ATCC 700358 / HUJEG-1	Actinomycetes
WP_071929043.1	476	GCF_001886715.1	*Nocardia mangyaensis* Y48	Actinomycetes
WP_106851818.1	486	GCF_003013675.1	*Rhodococcus hoagii* DSSKP-R-001	Actinomycetes
WP_131581136.1	488	GCF_004328705.1	*Rhodococcus* sp. ABRD24	Actinomycetes
WP_037313647.1	479	GCF_000023865.1	*Saccharomonospora viridis* DSM 43017	Actinomycetes
WP_127181560.1	485	GCF_003994395.1	*Streptomyces griseoviridis* F1-27	Actinomycetes
WP_125937754.1	479	GCF_003945505.1	*Streptomyces* sp. WAC06738	Actinomycetes
WP_066611676.1	491	GCF_001562255.1	*Bosea* sp. PAMC26642	Alphaproteobacteria

Table S3 (continued)

Protein accession	Protein length (aa)	RefSeq assembly accession	Organism/strain	Class
WP_106907122.1	497	GCF_000092285.1	*Caulobacter segnis* ATCC-21756	Alphaproteobacteria
WP_049797946.1	474	GCF_000165835.1	*Achromobacter xylosoxidans* A8	Betaproteobacteria
WP_013690625.1	491	GCF_000194745.1	*Burkholderia gladioli* BSR3	Betaproteobacteria
**APP18125.1**	484	GCF_052683555.1	***Burkholderia* sp. BC1**	Betaproteobacteria
WP_124686164.1	486	GCF_003854935.1	*Cupriavidus pauculus* FDAARGOS_614	Betaproteobacteria
WP_050409565.1	485	GCF_001191005.1	*Massilia* sp. NR4-1	Betaproteobacteria
WP_151050590.1	485	GCF_008807015.1	*Neisseria zalophi* ATCC BAA-2455	Betaproteobacteria
WP_013741981.1	491	GCF_000209655.1	*Pusillimonas* sp. T7-7	Betaproteobacteria
WP_023172286.1	506	GCF_000484535.1	*Gloeobacter kilaueensis* JS1	Cyanophyceae
WP_096298524.1	491	GCF_002327105.1	*Luteimonas chenhongjianii* 100111	Gammaproteobacteria
**NP_250815.1**	482	GCF_000006765.1	***Pseudomonas aeruginosa* PAO1**	Gammaproteobacteria
WP_124302839.1	484	GCF_003850405.1	*Pseudomonas chlororaphis* subsp. *piscium* SLPH10	Gammaproteobacteria
WP_011333787.1	485	GCF_000012445.1	*Pseudomonas fluorescens* Pf0-1	Gammaproteobacteria
WP_010459478.1	485	GCF_000257545.3	*Pseudomonas mandelii* JR-1	Gammaproteobacteria
WP_148118728.1	493	GCF_002155165.1	*Pseudomonas mendocina* NSYSU	Gammaproteobacteria
WP_011960017.1	492	GCF_000016885.1	*Psychrobacter* sp. PRwf-1	Gammaproteobacteria
WP_082746485.1	493	GCF_001700965.1	*Stenotrophomonas nitritireducens* 2001	Gammaproteobacteria
WP_149105814.1	493	GCF_008245145.1	*Olivibacter* sp. LS-1	Sphingobacteriia
**ALDH33B**				
WP_042444757.1	480	GCF_000010725.1	*Azospirillum* sp. B510	Alphaproteobacteria

Table S3 (continued)

Protein accession	Protein length (aa)	RefSeq assembly accession	Organism/strain	Class
WP_069053837.1	489	GCF_001713455.1	*Bosea* sp. RAC05	Alphaproteobacteria
WP_043872375.1	483	GCF_000819565.1	*Celeribacter indicus* P73	Alphaproteobacteria
WP_011582238.1	489	GCF_000014245.1	*Chelativorans* sp. BNC1	Alphaproteobacteria
WP_012187090.1	485	GCF_000018145.1	*Dinoroseobacter shibae* DFL 12 = DSM 16493	Alphaproteobacteria
WP_011456908.1	479	GCF_000013565.1	*Jannaschia* sp. CCS1	Alphaproteobacteria
WP_060847217.1	485	GCF_001548015.1	*Methylobacterium aquaticum* MA-22A	Alphaproteobacteria
WP_118852365.1	484	GCF_003491345.1	*Neorhizobium* sp. SOG26	Alphaproteobacteria
WP_118074832.1	481	GCF_003454795.1	*Novosphingobium* sp. THN1	Alphaproteobacteria
WP_076694628.1	483	GCF_001975705.1	*Pelagibaca abyssi* JLT2014	Alphaproteobacteria
WP_083716890.1	510	GCF_001975705.1	*Pelagibaca abyssi* JLT2014	Alphaproteobacteria
WP_014132963.1	480	GCF_000230555.1	*Pelagibacterium halotolerans* B2	Alphaproteobacteria
WP_041802429.1	484	GCF_000013745.1	*Rhodopseudomonas palustris* BisB18	Alphaproteobacteria
WP_015015887.1	489	GCF_000302535.1	*Acidovorax* sp. KKS102	Betaproteobacteria
WP_066344197.1	482	GCF_001676745.1	*Bordetella bronchialis* AU17976	Betaproteobacteria
WP_066350715.1	484	GCF_001676745.1	*Bordetella bronchialis* AU17976	Betaproteobacteria
WP_066659999.1	491	GCF_001676725.1	*Bordetella flabilis* AU10664	Betaproteobacteria
WP_086063627.1	482	GCF_002119685.1	*Bordetella genomosp*. 8 AU19157	Betaproteobacteria
WP_086067920.1	484	GCF_002119685.1	*Bordetella* genomosp. 8 AU19157	Betaproteobacteria
WP_043375884.1	485	GCF_000739375.1	*Comamonas testosteroni* TK102	Betaproteobacteria
WP_043376224.1	484	GCF_000739375.1	*Comamonas testosteroni* TK102	Betaproteobacteria

Table S3 (continued)

Protein accession	Protein length (aa)	RefSeq assembly accession	Organism/strain	Class
WP_133157140.1	489	GCF_004353865.1	*Hydrogenophaga pseudoflava* DSM 1084	Betaproteobacteria
WP_069047270.1	488	GCF_001713375.1	*Hydrogenophaga* sp. RAC07	Betaproteobacteria
WP_067756450.1	483	GCF_900089455.2	*Orrella dioscoreae* Orrdi1	Betaproteobacteria
WP_013093436.1	479	GCF_000092885.1	*Paraburkholderia atlantica* CCGE1002	Betaproteobacteria
WP_114157193.1	483	GCF_003330785.1	*Paraburkholderia graminis* PHS1	Betaproteobacteria
WP_124687682.1	485	GCF_003854895.1	*Pigmentiphaga* sp. H8	Betaproteobacteria
WP_124688010.1	483	GCF_003854895.1	*Pigmentiphaga* sp. H8	Betaproteobacteria
WP_102247273.1	486	GCF_002872475.1	*Pseudoazoarcus pumilus* SY39	Betaproteobacteria
WP_014995199.1	487	GCF_000300005.1	*Alcanivorax dieselolei* B5	Gammaproteobacteria
**ACA09737.1**	480	—	***Pseudomonas putida* Fu1**	Gammaproteobacteria
WP_025241912.1	480	GCF_000590475.1	*Pseudomonas stutzeri* 28a24	Gammaproteobacteria
**ALDH33C**				
WP_126424921.1	493	GCF_003963515.1	*Brevibacillus* sp. SCSIO07484	Bacilli
WP_062409776.1	488	GCF_001465255.1	*Paenibacillus naphthalenovorans* 32O-Y	Bacilli
